# Low-dose 2-deoxy glucose stabilises tolerogenic dendritic cells and generates potent in vivo immunosuppressive effects

**DOI:** 10.1007/s00018-020-03672-y

**Published:** 2020-10-19

**Authors:** M. Christofi, S. Le Sommer, C. Mölzer, I. P. Klaska, L. Kuffova, J. V. Forrester

**Affiliations:** 1 Institute of Medical Sciences, School of Medicine, Medical Sciences and Nutrition, Foresterhill, University of Aberdeen, Aberdeen, AB25 2ZD Scotland, UK; 2 Eye Clinic, Aberdeen Royal Infirmary, Aberdeen, Scotland UK; 3 Ocular Immunology Program, Centre for Ophthalmology and Visual Science, The University of Western Australia, Perth, WA Australia; 4 Centre for Experimental Immunology, Lions Eye Institute, Nedlands, WA Australia

**Keywords:** Cell therapy, Tolerance, Zbtb46, Autoimmunity, Metabolic programming

## Abstract

Cell therapies for autoimmune diseases using tolerogenic dendritic cells (tolDC) have been promisingly explored. A major stumbling block has been generating stable tolDC, with low risk of converting to mature immunogenic DC (mDC), exacerbating disease. mDC induction involves a metabolic shift to lactate production from oxidative phosphorylation (OXPHOS) and β-oxidation, the homeostatic energy source for resting DC. Inhibition of glycolysis through the administration of 2-deoxy glucose (2-DG) has been shown to prevent autoimmune disease experimentally but is not clinically feasible. We show here that treatment of mouse bone marrow-derived tolDC ex vivo with low-dose 2-DG (2.5 mM) (2-DGtolDC) induces a stable tolerogenic phenotype demonstrated by their failure to engage lactate production when challenged with mycobacterial antigen (Mtb). ~ 15% of 2-DGtolDC express low levels of MHC class II and 30% express CD86, while they are negative for CD40. 2-DGtolDC also express increased immune checkpoint molecules PDL-1 and SIRP-1α. Antigen-specific T cell proliferation is reduced in response to 2-DGtolDC in vitro. Mtb-stimulated 2-DGtolDC do not engage aerobic glycolysis but respond to challenge via increased OXPHOS. They also have decreased levels of p65 phosphorylation, with increased phosphorylation of the non-canonical p100 pathway. A stable tolDC phenotype is associated with sustained SIRP-1α phosphorylation and p85-AKT and PI3K signalling inhibition. Further, 2-DGtolDC preferentially secrete IL-10 rather than IL-12 upon Mtb-stimulation. Importantly, a single subcutaneous administration of 2-DGtolDC prevented experimental autoimmune uveoretinitis (EAU) in vivo. Inhibiting glycolysis of autologous tolDC prior to transfer may be a useful approach to providing stable tolDC therapy for autoimmune/immune-mediated diseases.

**Electronic supplementary material:**

The online version of this article (10.1007/s00018-020-03672-y) contains supplementary material, which is available to authorized users.

## Introduction

Dendritic cells [1] are a diverse, heterogeneous group of specialist immune cells that are the immune system’s primary professional antigen-presenting cells. Recent studies [2, 3] have described the various differentiation pathways of bone marrow progenitor DC from the time they enter the circulation as immature precursors (pre-DC) to when they populate the tissues as tissue-resident DC, and/or migrate to secondary lymphoid organs in a CCR7-dependent manner. During steady-state conditions, tissue-resident DC maintain immunological homeostasis by presenting self-antigen in the absence of co-stimulation. This ensures non-responsiveness to self-antigen by inducing T regulatory cells (T_REG_) or T cell anergy, in an NFκB-dependent mechanism [4], a process that is known as peripheral immune tolerance [5]. Dendritic cells also function to connect innate and adaptive immunity; when antigen presentation is coupled with “danger signals” (PAMPs, DAMPs), DC upregulate co-stimulatory molecules, and both, in conjunction, elicit an immune response. This dual function of tolerance induction by tolDC, and induction of immune responses by mature DC (mDC), makes using tolDC to promote immunological tolerance, an attractive prospect. Indeed, several pre-clinical studies, and Phase I/II clinical trials, have shown benefits in multiple diseases [6–9].

The harvesting and use of autologous tolDC as a therapeutic for the treatment of autoimmune disease and graft rejection has been explored in numerous studies [10–15]. Producing a sufficiently high yield of tolDC from either bone marrow (mouse), or circulating (human) precursors, via in vitro culture with granulocyte-monocyte colony-stimulating factor (GM-CSF) [16, 17] has been a difficult task for researchers. Several additional agents have been used to promote differentiation of tolDC, including vitamin D and dexamethasone (VitD/dex) [18–23], TGFβ and IL-10 [24], retinoic acid [25], LPS [26], monophosphoryl lipid A [27] and annexin-coated particles [28], all with similar efficacy. However, the stability of tolDC in vivo, could not be guaranteed [20], hence, this is currently considered the most important factor in bringing tolDC therapy to the clinic.

To date, three main DC subsets are recognised: conventional (c)DC1 (CD11c^int^CD11b^lo/neg^CD8a^+^CD103^+^), cDC2 (CD11c^hi^CD11b^hi^CD103^−^CD8a^−^) and plasmacytoid (p)DC (CD11c^−^CD11b^−^B220^+^SiglecH^+^) [29–32]. Standard GM-CSF bone marrow cultures result in a heterogeneous population of mostly cCD11b^+^ cDC2 and macrophages [30]. However, problematically, markers which characterise cDC2 are also expressed on other immune cells. Hence, refining protocols to simultaneously allow in-depth characterisation, and improved isolation of tolDC for in vivo use is a key challenge. To address this, and produce an enriched population of tolDC, we have developed a modified cell culture protocol [33] which includes characterisation of bone marrow tolDC using the specific DC transcription factor Zbtb46 [34, 35]. The procedure involves initial depletion of Lin^+^ cells and minimal handling/cell sorting, thus maximising the yield of tolDC and avoiding induction of immunogenic mDC.

Conversion of tolDC to mDC is accompanied by a shift from oxidative phosphorylation (OXPHOS/Krebs Cycle) and fatty acid oxidation to lactic acid production and reliance on glucose as the predominant carbon source [36] to generate ATP. Therefore, manipulation of these pathways to maintain DC OXPHOS and FAO has been proposed as a method to induce stable tolDC [37].

Previous studies have shown that the glucose analogue 2-DG, by competing for hexokinase [38] can block induction of mDC [39, 40] in vitro. Furthermore, 2-DG is effective in downregulating immune responses in vivo: treatment of mice with daily injections of 2-DG inhibited the development of experimental autoimmune neuritis (EAN) [41]. However, a daily injection of 2-DG as a treatment regime in humans is not practical or safe.

Here we show that both glycolysis and OXPHOS metabolism are temporarily blocked in resting tolDC treated with low-dose (2.5 mM) 2-DG (2-DGtolDC) in vitro in the presence of 11 mM glucose. 2-DGtolDC failed to engage aerobic glycolysis when challenged with mycobacterial antigen (Mtb) but instead activated OXPHOS metabolism and led to reduced NFkB signalling. In addition, PDL-1 and SIRP-1α checkpoint molecule expression were increased in 2-DGtolDC after Mtb challenge, while SIRP-1α dephosphorylation was prevented. We further show that a one-time adoptive transfer of 2-DGtolDC prevents induction of experimental autoimmune uveoretinitis (EAU) in mice.

## Materials and methods

### Animal studies

All animal procedures had been approved by the University of Aberdeen Ethics Review Committee Board and were performed under valid Project Licence (PPL60/4496) approved by the Home Office (UK) under the Animals (Scientific Procedures) Act 1986. C57BL/6 J and OT-II (DO11.10) mice were bred, maintained and housed at the Medical Research Facility, University of Aberdeen, UK. All mice were kept at 22 to 24 °C and 45–65% relative humidity on a 12 h light–dark cycle, with free access to standard chow diet and water.

### Induction of EAU and grading of disease

Experimental autoimmune uveoretinitis was induced in C57BL/6 J mice as previously described [26]. Briefly, mice were immunised with interphotoreceptor retinol-binding protein (IRBP) peptide 1–20 (GPTHLFQPSLVLDMAKVLLD) (New England Peptide, Gardner, MA, USA) (500 μg/mouse) emulsified in Complete Freund’s Adjuvant (CFA) containing 3.5 μg/ml of H37Ra (231131, Difco Laboratories, MI, USA) *Mycobacterium Tuberculosis* extract (Mtb), injected subcutaneously (s.c.) to the hind limbs. Pertussis toxin (PTx; Health Protection Agency, Chorley, UK) 1 μg/mouse was administered intraperitoneally (i.p.) at the same time. Clinical signs of disease (retinal vessel inflammation/vasculitis) were evaluated by fundoscopic examination using an otoscope light-based system as described [26]. Disease severity was graded using a clinical grading scale as described [42] with modifications [26]. Mice were humanely killed at stated times (see Results) using an approved Schedule One method under the Animals (Scientific Procedures) Act 1986. Eyes were harvested, fixed in 2.5% glutaraldehyde, embedded in resin, sectioned, and stained with haematoxylin and eosin (H&E). Sections (3 μm thick) of each globe were taken at several different levels. Disease severity was graded on a scale of 0–4 according to a semi‐quantitative histological scoring system as described previously [26, 43].

### Bone marrow-derived dendritic cell (BMDC) culture

Bone marrow was flushed from mouse hindlimb bones using RPMI-1640 GlutaMAX media (Gibco, Invitrogen, UK) containing 1% Pen/Strep. Erythrocytes were lysed using Red Blood Cell Lysing Buffer (Sigma, UK) as per the manufacturer’s instructions. T cells, B cells and MHC class II^+^ cells were depleted using sheep-anti-rat IgG coated Dynabeads (Invitrogen, Dynal, Oslo, Norway) and a cocktail of rat anti-mouse antibodies against CD4 (553727, BD Bioscience, UK), CD8 (550281 BD Bioscience, UK), B220 (550286, BD Bioscience, UK) and MHC class II (MCA09, Serotec, UK). The remaining cells were re-suspended in complete RPMI-1640 GlutaMAX media (Gibco) containing 5% heat-inactivated ultra-low IgG Foetal Calf Serum (Gibco), 1% Pen/Strep (Gibco), 1% NEAA (Gibco), 0.1% 2-mercaptoethanol (Gibco), 1% sodium pyruvate (Gibco) and 10 ng/ml GM-CSF (Miltenyi Biotec GmbH, Bergisch Gladbach, GER) (referred to as complete medium; cRPMI) and plated at 1.5 × 10^6^ cells in 2 ml per well in 12-well plates [day (d) 0 of culture]. Non-adherent floating cells were removed by gently exchanging 50% and 75% of the medium, respectively, for fresh cRPMI on d2 and d4 of culture. On d6 of culture, non-adherent cells were again removed by gentle pipetting and only the loosely adherent cell clusters were harvested. Those cells were then depleted of neutrophils and monocytes using IgG coated Dynabeads (Invitrogen) and anti-Ly6G/Ly6C (Gr-1) antibody (553122 BD Bioscience). The remaining cells were plated at 1.5 × 10^6^ in cRPMI (lacking sodium pyruvate) and cultured overnight prior to further use.

### BMDC treatments and stimulations

For in vivo experiments BMDC were cultured in the presence of 30 μg/ml IRBP 1–20 peptide (GPTHLFQPSLVLDMAKVLLD; New England Peptide, Gardner, MA) for 24 h with or without the presence of 2.5 mM 2-DG to induce an antigen‐specific response. For in vitro cell phenotype analysis BMDC were cultured for 24 h at various concentrations of 2-DG (Sigma, UK) and subsequently analysed by flow cytometry. For western blotting experiments, the medium exchange took place on d2 and d4 of culture. On d5 after Gr1^+^ cell depletion, BMDC were pre-treated with 2-DG (2.5 mM) for 3 h prior to incubation with LPS (1 μg/ml ultrapure lipopolysaccharide (LPS) (E. coli 0111:B4, catalog #tlrl-3pelps, InvivoGen, USA) for 1 h, or heat-inactivated Mtb extract (Mycobacterium Tuberculosis H37Ra, Difco (BD Bioscience, USA) (15 μg/ml) for 24 h.

### Enzyme-linked immunosorbent assay (ELISA)

Media supernatants over 2-DGtolDC cultures with and without Mtb-treatment were analysed for IL-10 and IL-12 concentrations using ELISA (R&D Systems Quantikine kits) according to the manufacturer`s instructions.

### T cell proliferation assay

CD4^+^ T cells from spleens and lymph nodes of DO11.10 (OT-II) mice were enriched by magnetic bead separation using a naïve CD4^+^ T cell isolation kit (#130–104-453, Miltenyi Biotec, GER), according to the manufacturer’s instructions. After isolation T cells were stained with carboxyfluorescein succinimidyl ester (CFSE) (2.5 μM/ml; Invitrogen, USA) and co-cultured with BMDC activated with 1 μg/ml LPS for 3 h before co-culture for 72 h in 96-well plates in the presence of ovalbumin (OVA) peptide 323–339 (#RP10610-5, GenScript, USA) at 1 μg/ml. After 72 h the cells were harvested and analysed by flow cytometry.

### Flow cytometry

Cells were incubated with fixable viability dye (eFluor 455UV; eBioscience, UK) followed by CD16/CD32 antibody (Fc-block; clone 2.4G2, BD Bioscience, UK), then stained with directly conjugated antibodies (10 μg/ml and analysed using an LSR II device (BD Bioscience, UK). The following antibodies were purchased from BD Bioscience, UK unless stated otherwise: MHC class II #553623, CD4 #552051, CD11b #550993, CD11c #550261, CD135 #562537, CD40 #562846, CD86 #560581, CD25 #553866, CCR7 #563596, PDL-1 #564716, Zbtb46 #565832, SIRP-1α #740159, c-kit #560185, CD115 #25–1152-82 (eBioscience) and CX3CR1 #149023 (Biolegend). Transcription factor buffer (BD Bioscience, USA, catalog #562574) was used in accordance with the manufacturer’s instructions to test for transcription factors. FlowJo software (TreeStar, Inc., Ashland, OR, USA) was used for data analysis.

### Metabolic studies

Cells were pre-treated with 2.5 mM 2-DG over 3 h, further treated with Mtb (as applicable/specified), harvested by resuspension in PBS, two centrifugation/wash steps (5 min, 300 g, 4 °C, PBS), and then homogenised/lysed at selected time points using the respective lysis buffer provided in each plate assay kit. The processed samples were centrifuged (5 min, 300 g, 4 °C) and supernatants collected in fresh tubes for analysis. The samples were tested for cellular uptake of glucose (cat no. ab102517), generation of pyruvate (cat no. ab65342), glucose-6-phosphate (G6P) (cat. no. ab83426), succinate (cat no. ab204718), and glycogen (cat no. ab65620), using colorimetric detection (Abcam, USA), performed as per the manufacturer’s instructions. Lactate dehydrogenase (cat no. ab102526) was quantified using a similar kit from the same provider.

### Immunoblotting

Cells were homogenised in radioimmunoprecipitation-assay (RIPA) buffer containing sodium-orthovanadate and protease with phosphatase inhibitors. Proteins were separated by SDS-PAGE 4–12% gradient precast gels (Invitrogen, UK) and transferred to nitrocellulose membranes. All antibodies used for immunoblotting were purchased from Cell Signalling Technology, MA, USA (unless otherwise stated): #8954 phospho-tyrosine, #13379 SIRP-1α (pTyr-SIRP-1α), #4228 phospho-PI3 kinase (PI3K) p85 (Tyr458), #4257 PI3K p85, #4060 phospho-AKT (Ser473), #4691 AKT, #3033 phospho-NFκB p65 (Ser536), #8242 NFκB p65, #4810 phospho-NFκB2 p100 (Ser866/870), #4882 NFκB2 p100/p52, #5174 GAPDH. Immunoblots were visualised using enhanced chemiluminescence (PeqLab) and quantified using ImageJ.

### Data analysis

Data are presented as mean ± SEM unless otherwise stated. Statistical analyses were conducted using one/two-way ANOVA, followed by Tukey *post-hoc* test using the GraphPad Prism 7 software (GraphPad Software). Statistical significance was assumed based on a 95% level of confidence.

## Results

### Altering the GM-CSF bone marrow culture protocol to include depletion of Lin^+^ cells produces an enriched tolDC population

Protocols for preparation of mouse BMDC have mostly aimed at high yield production of mDC as “cell vaccines” for therapy of cancer and infectious disease. Helft et al*.* selected MHC class II^hi^CD11c^hi^ cells – classical markers of mDC [30] – to study GM-CSF cultured BMDC by flow cytometry, while Jin et al*.* maximised the yield of MHC class II^hi^ mDC by varying BMDC density in culture [44]. In both studies, there was significant “contamination” with non-DC myeloid cells, identified as monocyte/macrophage type cells as well as myeloid precursor (MDP) cells. Few similar detailed phenotypic analyses of murine tolDC preparations have been performed [33, 44–46]. In part, this may be due to low expression of MHC class II by tolDC, hence MHC class II expression cannot be used to accurately identify tolDC populations. BMDC in the mouse are predominantly CD11b^hi^CD11c^hi^ cDC2 [29, 47] and leave the bone marrow to enter the circulation as pre-DC, prior to populating the tissues and differentiating as either CD4^+^ or CD8^+^ tissue-resident DC. Therefore, we used CD11b and CD11c expression for initial identification of BMDC.

The following is a phenotypic analysis of the total cell population in the bone marrow culture as prepared above and which was used in the studies described below. This preparation, which we term “tolDC”, was obtained after lysis of red blood cells, depletion of Lin^+^ differentiated cells, followed by harvesting loosely adherent cell clusters and further depletion of Gr-1^+^ cells (neutrophils, and inflammatory monocyte-DC) from the culture at d6 [33]. No additional steps were taken in cell purification since it was recognised that this would reduce the overall yield of tolDC as well as lead to unwanted activation of the DC population, as detailed in the Introduction.

**Fig. 1:**
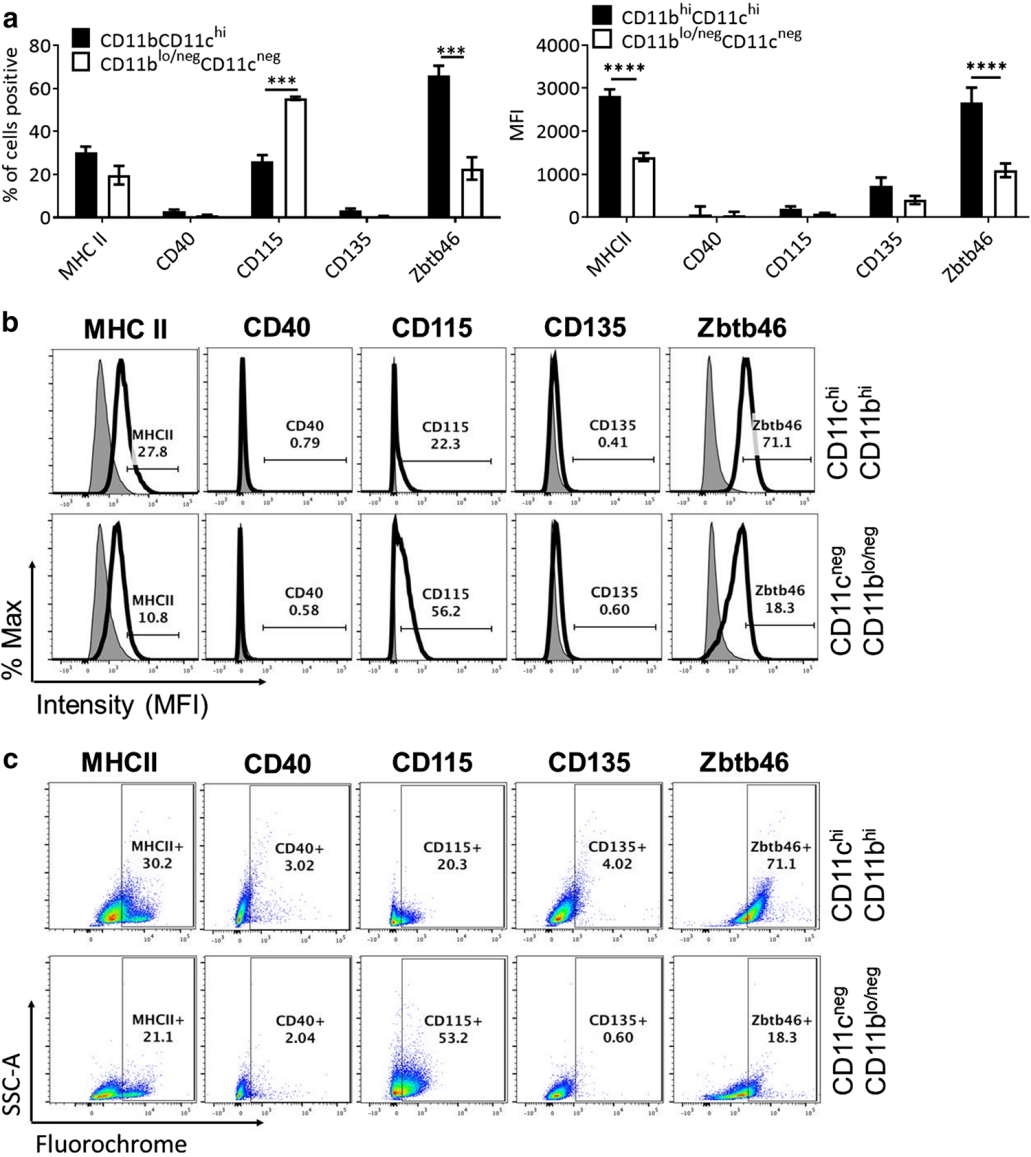
*Lin*^+^ *depletion of GM-CSF bone marrow cultures generates an enhanced yield of resting tolerogenic (tol)DC.* Cells were cultured as described in Methods and loosely adherent clusters harvested on d6 following seeding on d0. Flow cytometry analysis of live, single CD11b^hi^CD11c^hi^ or remaining cells: **a** percentage of cells positive for MHC Class II (graph on left) and Mean Fluorescent Intensity (MFI) values for MHC Class II (graph on right), CD40, CD115 and CD135 surface markers, and the transcription factor Zbtb46 gated on live, single CD11b^hi^CD11c^hi^ cells (black bar) and CD11b^lo/neg^CD11c^neg^ (white bar). **b** Representative corresponding MFI histograms for: MHC II, CD40, CD115, CD135, and Zbtb46, gated on live, single CD11b^hi^CD11c^hi^ cells (top row) and CD11b^lo/neg^CD11c^neg^ (bottom row). **c** Representative flow cytometry dot plots for: MHC II, CD40, CD115, CD135, and Zbtb46, gated on live, single CD11b^hi^CD11c^hi^ cells (top row) and CD11b^lo/neg^CD11c^neg^ (bottom row). Error bars denote Standard Error of the Mean (SEM) with *p* values: *** < 0.001, **** < 0.0001, *n* = 4

The preparation of tolDC contained an enriched population of CD11b^hi^CD11c^hi^ cells (~ 55% of the total population) (Supp. Figure 1d) of which a proportion (~ 30%) expressed low/intermediate levels of MHC class II (Fig. 1a-c) as described previously [33]. There were no MHC class II^hi^ cells. In addition, the CD11b^hi^CD11c^hi^ cell population expressed minimal levels of CD40 and CD86 and were mostly negative for the DC differentiation marker CD135 (Fig. 1, Supp. Figure 2). Importantly, 66% of the cells in the CD11b^hi^CD11c^hi^ fraction were + ve for the definitive DC marker Zbtb46. A proportion of the non-CD11b^hi^CD11c^hi^ cell fraction, which consisted of CD11b^lo/neg^CD11c^neg^ cells (Supp. Figure 1d) also expressed Zbtb46 (~ 20%) (Fig. 1a-c). CD11b^lo/neg^CD11c^neg^ cells also expressed minimal levels of CD40 and CD86 and were mostly negative for the DC differentiation marker CD135 (Fig. 1, Supp. Figure 2). However, 56% of the CD11b^lo/neg^CD11c^neg^ cells (~ 25% of total tolDC preparation) expressed CD115, albeit at very low levels, indicating that these cells were at an earlier stage of myeloid cell development possibly at the MDP progenitor stage. CD11b^lo/neg^CD11c^neg^ cells were negative for the early haematopoietic stem cell (HSC) and for the GMP/MDP marker c-kit but contained a high percentage of the pre-DC marker CX3CR1 although at similar MFI to the CD11b^hi^CD11c^hi^ population (Supp. Figure 3). Overall, it was difficult to precisely define the developmental stage of the CD11b^lo/neg^CD11c^neg^ but it is likely that they contained a mixture of progenitor/pre-DC and pre-monocyte/macrophage. This is consistent with similar studies of GM-CSF-based preparation of immunogenic DC in which MHC Class II was used to identify DC and in which there was a significant proportion of monocyte/macrophage precursor cells [30, 44]. In summary, the CD11b^hi^CD11c^hi^ cell fraction of the tolDC preparation contained on average 66% Zbtb46^+^ *bona fide* DC, while the final concentration of Zbtb46^+^ DC in the tolDC preparation was ~ 45%. The Zbtb46 negative cells contained varying proportions of myeloid cells at different stages of development.

### Low-dose (2.5 mM) 2-DG does not affect tolDC viability

As indicated above, while tolDC have been investigated for their use in treating disease, previous work has found that they can be unstable and liable to activation (“maturation”), thus converting to mDC [48], likely due to exposure to pro-inflammatory in vivo microenvironments. Since glycolysis underpins DC activation [39, 49] we investigated whether inhibition of glycolysis via 2-DG can stabilise tolDC and prevent their conversion to mDC when challenged.

Given that glucose is essential for DC survival, we first evaluated the effects of 2-DG on DC viability cultured in the presence of glucose (Supp. Figure 4). We found that high-dose (10 mM) 2-DG treatment led to apoptosis in ~ 22% of DC (*p* < 0.05) while low doses (1 mM and 2.5 mM) had no significant impact on the number of cells becoming apoptotic (Supp. Figure 4a, c). Furthermore, stimulation with Mtb (15 µg/ml) had no significant effect (Supp. Figure 4a, c). As expected, cells cultured in the absence of glucose became apoptotic at all doses (Supp. Figure 4b, d). Based on these results, we selected a dose of 2.5 mM 2-DG in glucose-containing media (11 mM) for all later experiments.

### Low-dose 2-DG treatment enhances tolDC phenotype

**Fig. 2:**
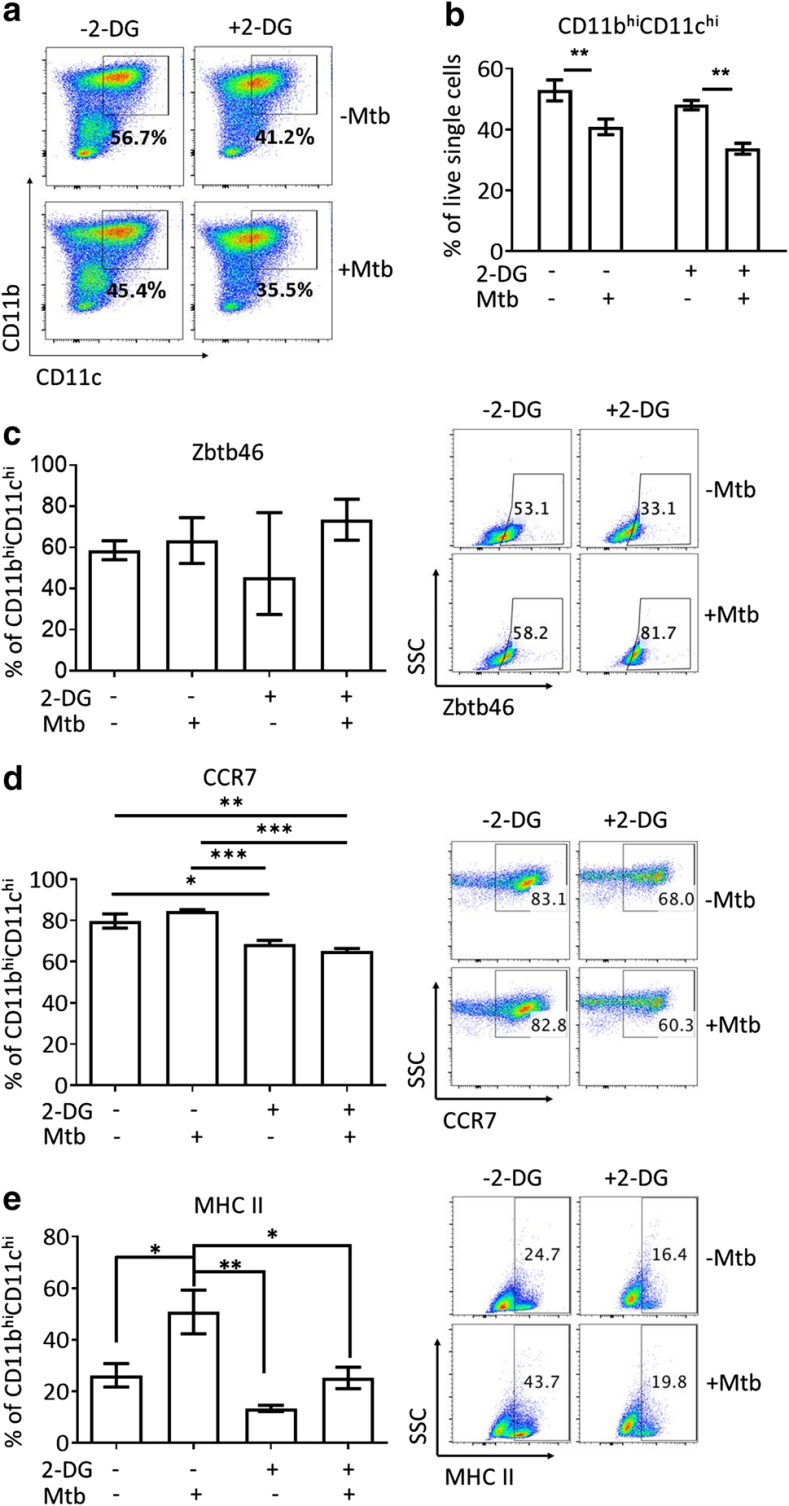
*Treatment of bone marrow tolerogenic DC (tolDC) with 2-deoxy glucose (2-DG) results in an enhanced tolerogenic DC phenotype (2-DGtolDC)*. **a** BMtolDC were cultured as described and treated with 2-DG (2.5 mM) (top panels), and/or stimulated with 15 µg/ml heat inactivated mycobacterial extract, Mtb (bottom panels); representative plots of CD11b vs. CD11c (single, live cells) gating on CD11b^hi^CD11c^hi^ cells and showing the percentage cell populations present within this gate; **b** histograms of percentage gated single live CD11b^hi^CD11c^hi^ cells as in Fig. 2a with or without 2-DG treatment and/or Mtb stimulation (*n* = 5); **c** the percentage of Zbtb46^+^ cells in the gated CD11b^hi^CD11c^hi^ population after treatment with 2-DG and/or stimulation with Mtb (*n* = 6) (left); representative dot plots of Zbtb46 gated on CD11b^hi^CD11c^hi^ population (right); **d** expression of the chemokine receptor CCR7 on gated CD11b^hi^CD11c^hi^ was significantly reduced after treatment with 2-DG (*p* < 0.001, *n* = 3), but unaffected by Mtb stimulation; representative dot plots of CCR7 gated on CD11b^hi^CD11c^hi^ population (right); **e** gated CD11b^hi^CD11c^hi^ cells exposed to Mtb alone increased expression of MHC class II as expected; in contrast, 2-DG treatment reduced expression of MHC Class II on untreated and Mtb-treated CD11b^hi^CD11c^hi^ cells; representative dot plots of MHC Class II gated on CD11b^hi^CD11c^hi^ population (right); (*p* values: * < 0.05, ** < 0.01, *** < 0.001 *n* = 4). Error bars in **b**-**e** denote Standard Error of the Mean (SEM)

Since the greater proportion of *bona fide* DC were contained in the CD11b^hi^CD11c^hi^ fraction of the tolDC preparation (Fig. 1) the following analysis reports on the effects of 2-DG on this fraction. Cells prepared in standard glucose-containing (11 mM) cRPMI (see Methods) were treated on d6 of culture with 2.5 mM 2-DG for 24 h. This resulted in a 15% reduction in CD11b^hi^CD11c^hi^-expressing cells (Fig. 2a, b). Similarly, there was a non-significant reduction in Zbtb46^+^ cells in the CD11b^hi^CD11c^hi^ cells to an average of 40% which increased after additional stimulation with Mtb to ~ 70% (Fig. 2c). In contrast, 2-DG treatment reduced the percentage of CCR7^+^ cells from ~ 80% to ~ 70% in both the Mtb-treated and -untreated CD11b^hi^CD11c^hi^ fraction of the tolDC preparation (Fig. 2d). As discussed above, MHC class II is a core DC marker and the level of surface expression is reflective of the cells' activation status. A proportion of CD11b^hi^CD11c^hi^ cells (~ 25%) expressed low/intermediate levels of MHC class II rising to 50% following Mtb stimulation (Fig. 2e). Importantly, 2-DG significantly reduced MHC class II expression in both the unstimulated (~ 15% average) and Mtb-stimulated (~ 22% average) CD11b^hi^CD11c^hi^ fraction (Fig. 2e) and prevented the Mtb-mediated upregulation of CD86 (Supp. Figure 2) while maintaining high levels of CX3CR1 (Supp. Figure 3c).

Our data suggest that the CD11b^hi^CD11c^hi^ cells in the GM-CSF BMDC culture represent mostly a mix of MHC Class II^lo/neg^ DC and CX3CR1^+^ pre-DC typical of new entrants to the pool of circulating DC [50, 51]. Furthermore, we demonstrate that 2.5 mM 2-DG treatment of Lin^+^-depleted, GM-CSF-cultured, Gr-1^+^ depleted BMDC cells yields an enriched population of stable tolDC containing ~ 40–60% Zbtb46^+^, ~ 70% CCR7^+^MHC class II^lo/neg^ cells.

### Antigen-specific T cell proliferation in response to 2-DGtolDC is reduced

**Fig. 3:**
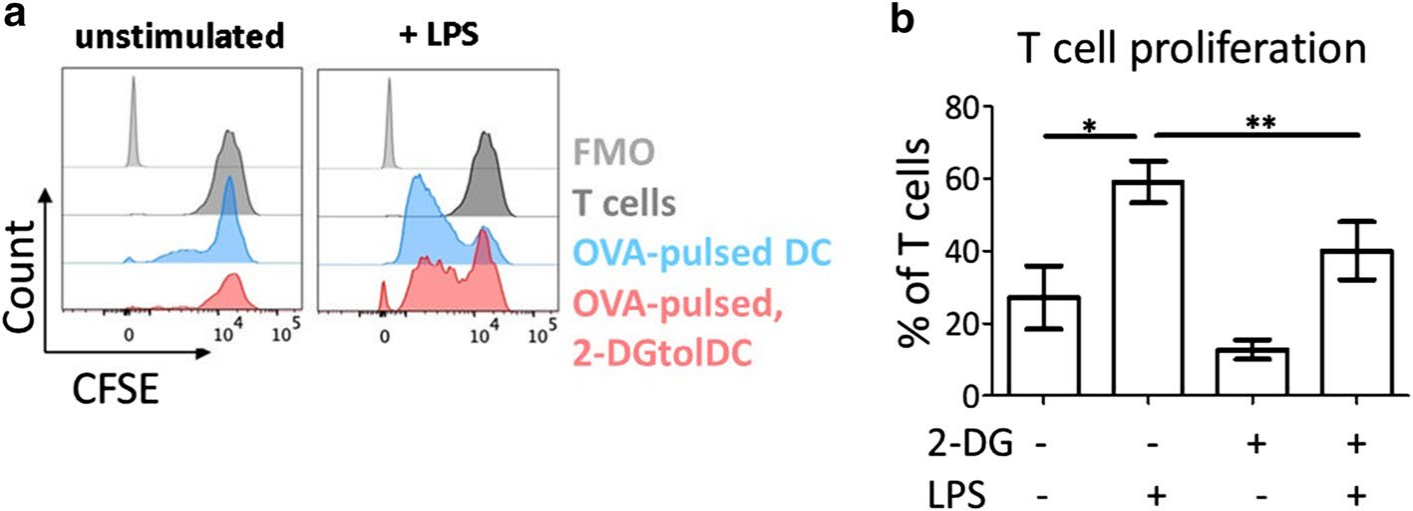
*LPS-activated, 2-DG-treated DC (2-DGtolDC) are poor stimulators of T cell proliferation*. Ovalbumin (OVA peptide)-specific OT-II cells were stimulated by OVA-pulsed 2-DG-treated and control untreated DC, with or without prior activation with LPS (1 µg/ml) and T cell proliferation assessed using the CFSE dilution assay as described in Methods. Cells were harvested from the co-cultures after 72 h and assessed for T cell proliferation by flow cytometry, gating on the T cell population. **a** Representative flow cytometry histograms of T cell proliferation. T cells cultured in isolation, did not proliferate (black shading), while T cells co-cultured with LPS-activated, OVA-pulsed DC induced robust proliferative responses (blue shading). In contrast, T cells co-cultured with 2-DG-treated OVA-pulsed DC (red shading) proliferated only moderately compared to co-culture with untreated DC. **b** Quantitative analysis of T cell proliferation measured by CFSE dilution assay. Treatment of BMDC with 2-DG significantly reduced the induction of T cell proliferation by LPS-activated BMDC (*p < 0.05, **p < 0.01, *n* = 3)

We next wished to assess the T cell activation potential of 2-DGtolDC. Thus, we examined T cell proliferation using the OT-II transgenic mouse model which contains an enriched population of CD4^+^ T cells expressing a TCR specific for the OVA 323–339 peptide (ISQAVHAAHAEINEAGR) [52]. T cells from lymph nodes and spleens of OT-II mice were labelled with CFSE and co-incubated with tolDC or 2-DGtolDC pulsed with OVA 323–339 peptide (1 µg/ml) and activated by LPS stimulation (1 µg/ml for 3 h). T cells were harvested after 72 h of co-culture and assessed for proliferation. Unstimulated tolDC induced moderate levels of T cell proliferation (27.1%) which increased on activation of the DC with LPS (59.0%). In contrast, 2-DGtolDC failed to induce significant T cell proliferation over baseline levels of T cells cultured without BMDC, even when they were activated with LPS (Fig. 3a, b).

### TolDC surface expression of PDL-1 and SIRP-1α is increased by 2-DG treatment

**Fig. 4:**
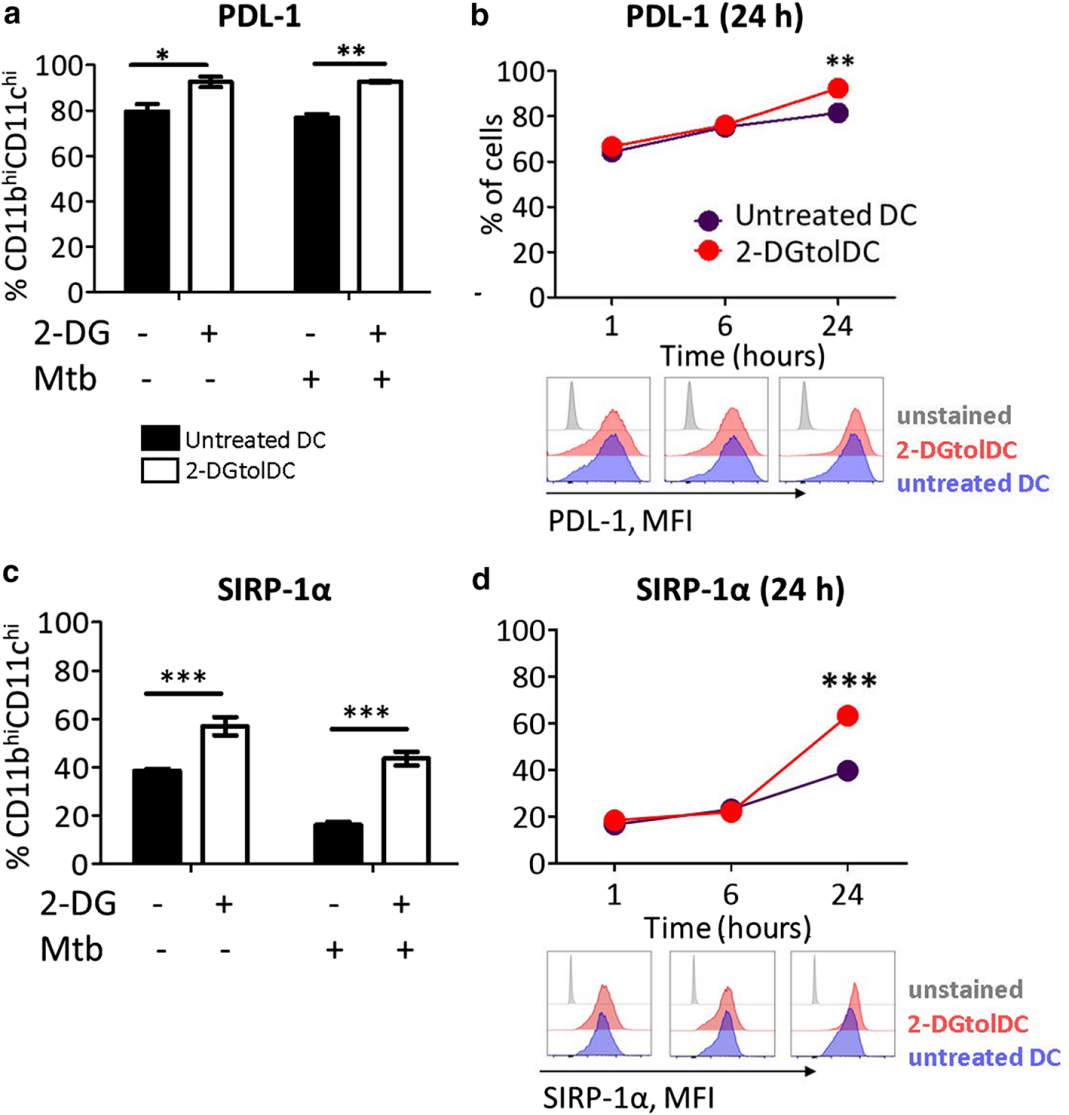
*Expression of PDL-1 and SIRP-1α are increased in DC treated with 2-DG (2-DGtolDC)*. **a** Percentage of PDL-1^+^ DC, with or without 2-DG (2.5 mM over 24 h), and/or, stimulated with heat-inactivated mycobacterial extract (Mtb, 15 μg/ml over 24 h) (gated on live, singlet, CD11b^hi^CD11c^hi^ cells). Treatments are indicated by the ± matrix below graph. **b** Percentage of PDL-1^+^ DC treated with 2-DG for 1/6/24 h. Below, histograms of PDL-1 expression intensity (MFI) for each time point are shown, with untreated DC in blue, and 2-DGtolDC in red. Note increase in the intensity of PDL-1 expression in 2-DGtolDC. **c** Percentage of DC in live, singlet CD11b^hi^CD11c^hi^ gate, that are positive for SIRP-1α, DC treatments/stimulations are indicated in the ± matrix. **d** Percentage of DC, treated with 2-DG for 1/6/24 h, positive for SIRP-1α. Below, histograms of SIRP-1α MFI for each time point are shown, with untreated DC in blue, and 2-DGtolDC in red. Error bars denote Standard Error of the Mean (SEM), *p* values: * < 0.05, ** < 0.01, *** < 0.001, *n* = 3

The binding of PDL-1 on DC to PD-1 on T cells classically transmits an inhibitory signal, leading to reduction of antigen-specific T cell proliferation [53]. Given that 2-DGtolDC fail to induce appropriate proliferation of antigen-specific T cells we evaluated the expression of PDL-1 on 2-DGtolDC. Untreated tolDC expressed high levels of PDL-1 positivity both by cell number and MFI. These were further increased by 2-DG treatment (2.5 mM, 24 h) (93% vs. 82%, respectively, *p* < 0.05), a difference which was more pronounced after stimulation with Mtb (Fig. 4a, b).

We also investigated the expression of a second immune checkpoint molecule, SIRP-1α [54], a known negative regulator of DC maturation [55] and mediator of DC and T cell homeostasis [56–58]. Flow cytometric analysis of surface SIRP-1α revealed that treatment with 2-DG increased the number of cells expressing SIRP-1α from 39 to 57% (*p* < 0.001). In line with published literature, SIRP-1α surface expression declined with Mtb stimulation but remained elevated in 2-DG treated cells (16% and 43.5% positive cells, respectively) (Fig. 4c). As for PDL-1 we conducted a time course of 2-DG treatment, finding that there was no increase in the percentage of cells expressing surface SIRP-1α in the first 6 h post-2-DG presence (in the absence of Mtb stimulation). However, this was significantly increased at 24 h post-treatment, with 39% of untreated cells and 63% of 2-DG-treated cells expressing SIRP-1α (p < 0.001) (Fig. 4d). In addition, as for PDL-1, there was an increase in SIRP-1α MFI from 1 h.

### Low-dose 2-DGtolDC produce less lactate at rest and shift to OXPHOS metabolism after stimulation with Mtb

**Fig. 5:**
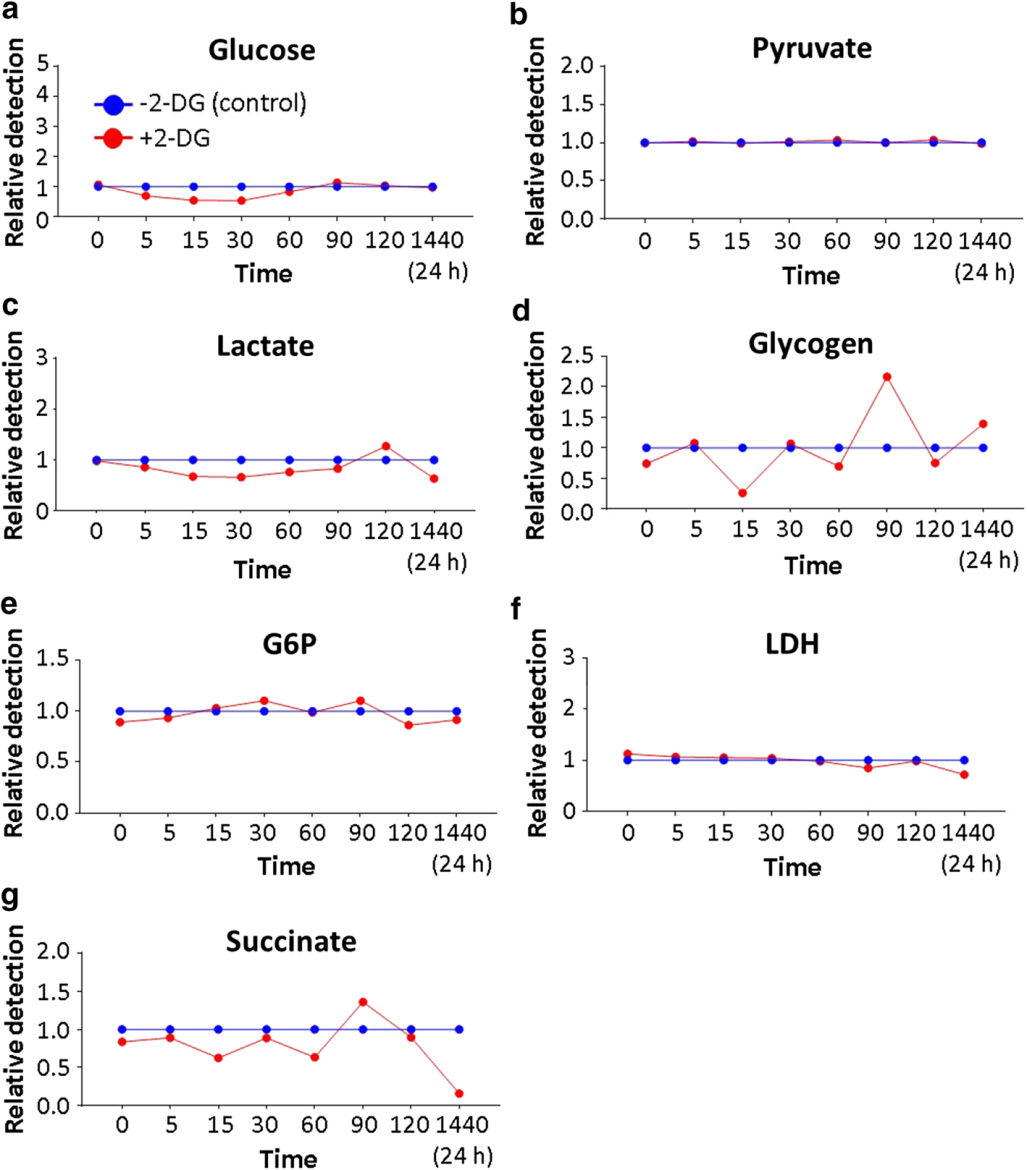
*Resting 2-DGtolDC have reduced levels of anaerobic glycolysis and OXPHOS metabolism*. Graphs show the relative levels of intracellular glucose and glucose metabolites between untreated and 2-DG-treated cells. BMDC were prepared as described, treated with 2.5 mM 2-DG for 3 h, and lysed at different time points [0, 5, 15, 30, 60, 90, 120 min and 1440 min (24 h) post-treatment] and tested with the respective plate assay kit (see Methods). Control cells were not treated with 2-DG. The relative levels of the various metabolites in control cells (blue lines) are shown in comparison with 2-DG-treated cells (red lines): **a** after 15 min 2-DGtolDC contained ~ 45% less glucose than untreated DC; **b** no difference was observed in pyruvate levels; **c** a ~ 40% reduction in lactate was observed peaking at 30 min and a late increase in lactate levels was noted at 120 min 2-DGtolDC; **d** glycogen levels dipped (15 min, 60 min) and rose (90 min) in 2-DGtolDC compared to controls; **e** and **f** there was no significant change in G6P and LDH levels between 2-DG-treated and -untreated DC; **g** early (15 min, 60 min) reductions in succinate levels with a later (90 min) rise followed by a marked late fall (24 h) were observed in 2-DGtolDC compared to untreated DC (all data from three replicates at each time point)

Tolerogenic DC were incubated with 2.5 mM 2-DG in the presence of 11 mM glucose for 3 h and sequential cell lysates prepared for a time-course analysis of intracellular glucose and glucose metabolic products. In resting tolDC, treatment with 2-DG, caused up to 50% reduction in glucose uptake which reached its lowest level 15 min after exposure to 2-DG, returning to normal levels by 90 min (Fig. 5a). There was a corresponding reduction in lactate levels up to 90 min with a brief rise after 120 min (Fig. 5c). No significant changes in pyruvate, glucose-6-phosphate (G6P), or lactate dehydrogenase were observed during this early exposure to 2-DG (Fig. 5b, e, f). There was a late reduction in lactate by 24 h (Fig. 5c). Reduced levels of succinate were observed at 15 and 60 min with a brief rise at 90 min. By 24 h, intracellular succinate levels had declined almost to nil (Fig. 5g). There were also large swings in intracellular glycogen levels in 2-DGtolDC with a decline almost to nil at 15 min, a second small drop at 60 min and a sharp rise 90 min after exposure of tolDC to 2-DG (Fig. 5d). After 24 h, glycogen levels remained elevated in 2-DGtolDC.

These data suggest that both anaerobic glycolysis (*vide* lactate and LDH) and OXPHOS (*vide* succinate) metabolism are inhibited in low-dose resting 2-DGtolDC. In the case of anaerobic glycolysis, the effects lasted for 90 min. However, OXPHOS metabolism remained significantly reduced long after the effects of 2-DG had disappeared. In addition, the large swings in intracellular glycogen levels suggest that glycogen stores may provide fuel for cellular homeostasis during 2-DG-induced periods of relative glucose starvation.

**Fig. 6:**
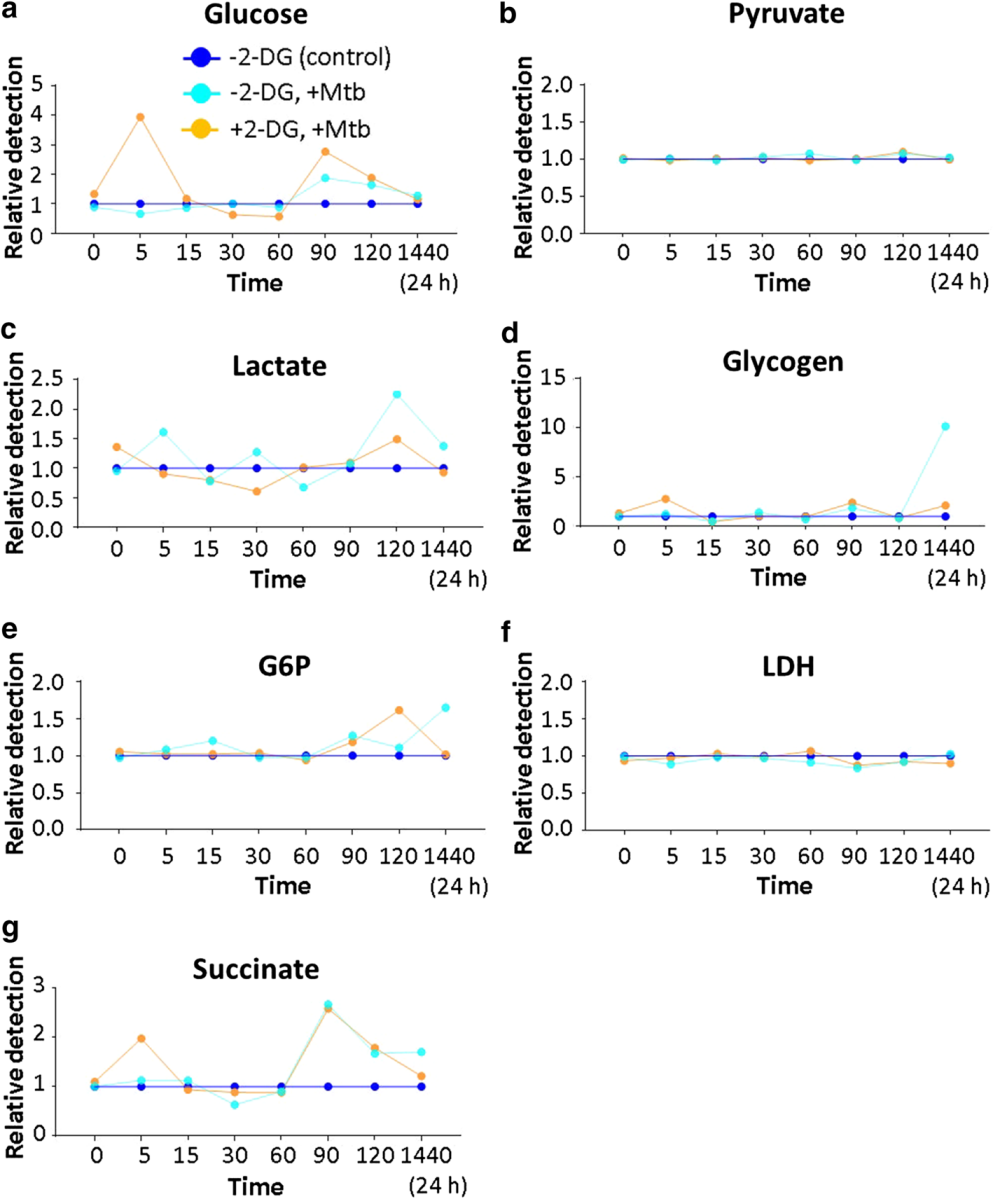
*Mtb-stimulated 2-DGtolDC fail to activate anaerobic metabolism but engage OXPHOS*. Graphs show the relative difference in intracellular glucose and glucose metabolites between tolDC (light blue line) and 2-DGtolDC (yellow line) after stimulation with heat-inactivated mycobacterial extract (Mtb, 15 µg/ml). Dark blue line (baseline control) represents unstimulated non-2DG-treated control DC. **a** 2-DGtolDC showed a marked rise in intracellular glucose levels 5 min after Mtb stimulation which declined between 30 and 60 min and peaked again at 90 min; non-2-DG-treated DC showed a smaller late rise in glucose uptake at 90 min after Mtb stimulation; **b** there was no difference in pyruvate levels; **c** non-2DG-treated DC showed increases in lactate at 5, 30 and 120 min while 2-DGtolDC had a reduced level at 30 min and a late rise at 120 min; **d** 2-DGtolDC showed small rises in glycogen at 5 and 90 min while non-2-DG-treated DC had a marked late increase (~ × 10); **e** G6P was increased at 120 min in 2-DGtolDC and at 24 h in non-2-DG-treated DC after Mtb stimulation; G6P levels were increased in 2-DGtolDC 120 min after exposure to 2-DG; **f** there was no difference in LDH levels; **g** there was an early increase (5 min) in succinate levels in 2-DGtolDC compared to non-2-DG-treated DC after Mtb stimulation while both 2-DG-treated and non-2-DG-treated DC had a similar marked late increase in intracellular succinate levels (90–120 min). Measurements were done in triplicate

Glycolysis and OXPHOS metabolism were markedly different in Mtb-stimulated 2-DGtolDC. High levels of intracellular glucose were observed in 2-DGtolDC peaking 5 min after stimulation with Mtb and again at 90 min, suggesting an accumulation of unmetabolized glucose (Fig. 6a). Lactate production declined during the first 30 min but returned to baseline by 60 min and showed a small increase by 120 min before normalising once more by 24 h (Fig. 6c). This contrasted with non-2DG-treated cells which generated peaks of lactate production 5 min, 30 min and 120 min after Mtb stimulation (Fig. 6c). Lactate dehydrogenase levels remained unchanged throughout the period of observation (Fig. 6f). No early changes were noted in pyruvate or G6P levels in 2-DGtolDC although there was a late rise at 2 h in G6P levels, returning to normal by 24 h (Fig. 6e). Interestingly, there were two brief peaks in glycogen levels at 5 and 90 min in 2-DGtolDC but the late surge in glycogen seen in Mtb-stimulated non-2-DG-treated cells was not observed (Fig. 6d). There were also two peaks of increased succinate levels, in 2-DGtolDC after Mtb stimulation, one at 5 min after exposure to 2-DG and a second at 90 min which coincided exactly with a similar peak in Mtb-stimulated non-2-DG-treated cells.

These data indicate that Mtb stimulation of 2-DGtolDC in the presence of glucose failed to initiate expected levels of anaerobic glycolysis. Instead, glucose appeared to accumulate in the cell at 5 min and glycogen stores increased. Interestingly, unlike in resting cells, the early surge in OXPHOS metabolism (increase in succinate, 5 min) after Mtb stimulation suggests that relative glucose starvation due to 2-DG prevented Mtb-induced anaerobic glycolysis but allowed OXPHOS metabolism to proceed. The second increase in succinate in 2-DGtolDC at 90 min coincided with a similar increase in non-2-DG Mtb-stimulated cells indicating that the early effects of 2-DG had died out, an interpretation of the data which was also supported by the late rise in G6P.

The data thus suggest that bone marrow pre-DC/tolDC as prepared here, and cultured in 11 mM glucose with 2.5 mM 2-DG, convert to a stable tolDC phenotype, in which anaerobic glycolysis (*vide* lactate production) is blocked while OXPHOS metabolism (*vide* succinate production) is increased when the cells are challenged with Mtb protein. This altered metabolic phenotype coincides with the changes in DC surface markers and cell function as well as the surface expression of checkpoint molecules induced by 2-DG as described in the previous sections. Low-dose 2-DG appears to re-programme resting tolDC to respond with an early shutdown of anaerobic glycolysis and a shift to OXPHOS metabolism when challenged with PAMPs such as Mtb and LPS (see below).

### 2-DG suppresses PI3K-AKT and canonical NFκB signalling and enhances non-canonical NFκB signalling in tolDC

**Fig. 7:**
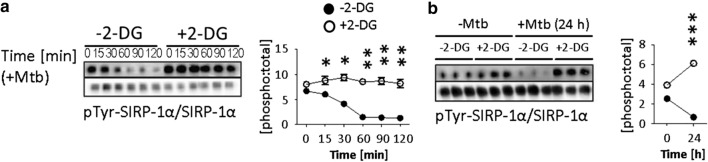
*2-DGtolDC fail to dephosphorylate SIRP-1α after Mtb stimulation*. Lin^+^-depleted bone marrow tolerogenic DC (BMtolDC) were cultured for 5 days in GM-CSF-containing cRPMI media (10 ng/ml), Gr-1^+^ cells were depleted, and the tolDC cultured on d6 for 3 h with or without 2.5 mM 2-DG. They were then stimulated with heat-inactivated mycobacterial extract (Mtb, 15 μg/ml) for the time shown (0–120 min, or 0–24 h) and assessed for SIRP-1α. **a** Immunoblots of tyrosine phosphorylated and total SIRP-1α, 0–120 min. Phosphorylation was evaluated by immunoprecipitation (IP) of phospho-tyrosine (pTyr) proteins and immunoblotting with total SIRP-1α protein; below, pre-IP samples probed for SIRP-1α total protein are shown to demonstrate equal input into IP. Quantification of pTyr-SIRP-1α values are presented as the [phospho:total] ratio; 2-DGtolDC are shown in open circles, untreated DC in filled circles. Times refer to post-Mtb stimulation. **b** pTyr-SIRP-1α (measured by pTyr IP), at 0 and 24 h with and without Mtb stimulation (*n* = 3 for each condition). 2-DG prevented the downregulation of pTyr-SIRP-1α induced by Mtb stimulation at 24 h in BMDC. Quantification of pTyr-SIRP-1α values shown as for **a**; Immunoblots were visualised using enhanced chemiluminescence (PeqLab) and quantified using ImageJ

Phosphorylated SIRP-1α (pSIRP-1α) is known to sequester PI3K signalling in myeloid cells during TLR stimulation [59] and to negatively regulate DC maturation [55]. We explored the possibility of direct effects of 2-DG on cell signalling, through this mechanism (Fig. 7). Tolerogenic DC were pre-treated with 2-DG (3 h, 2.5 mM), and then stimulated with Mtb for up to 2 h (Fig. 7a) or for 24 h (Fig. 7b), following which pTyr-SIRP-1α was assessed by pTyr-immunoprecipitation, and western blotting for total SIRP-1α. SIRP-1α is constitutively phosphorylated in tolDC (see time point zero in Fig. 7a). In non-2-DG-treated tolDC, SIRP-1α is rapidly dephosphorylated within the first two hours following Mtb stimulation. In contrast, 2-DG-treated tolDC challenged with Mtb retain their pTyr-SIRP-1α levels (p < 0.001) (Fig. 7a). Furthermore, this effect is not only sustained but increased up to at least 24 h (*p* < 0.001), by which time non-2-DG-treated tolDC have extremely low levels of pTyr-SIRP-1α after Mtb stimulation (Fig. 7b).

**Fig. 8:**
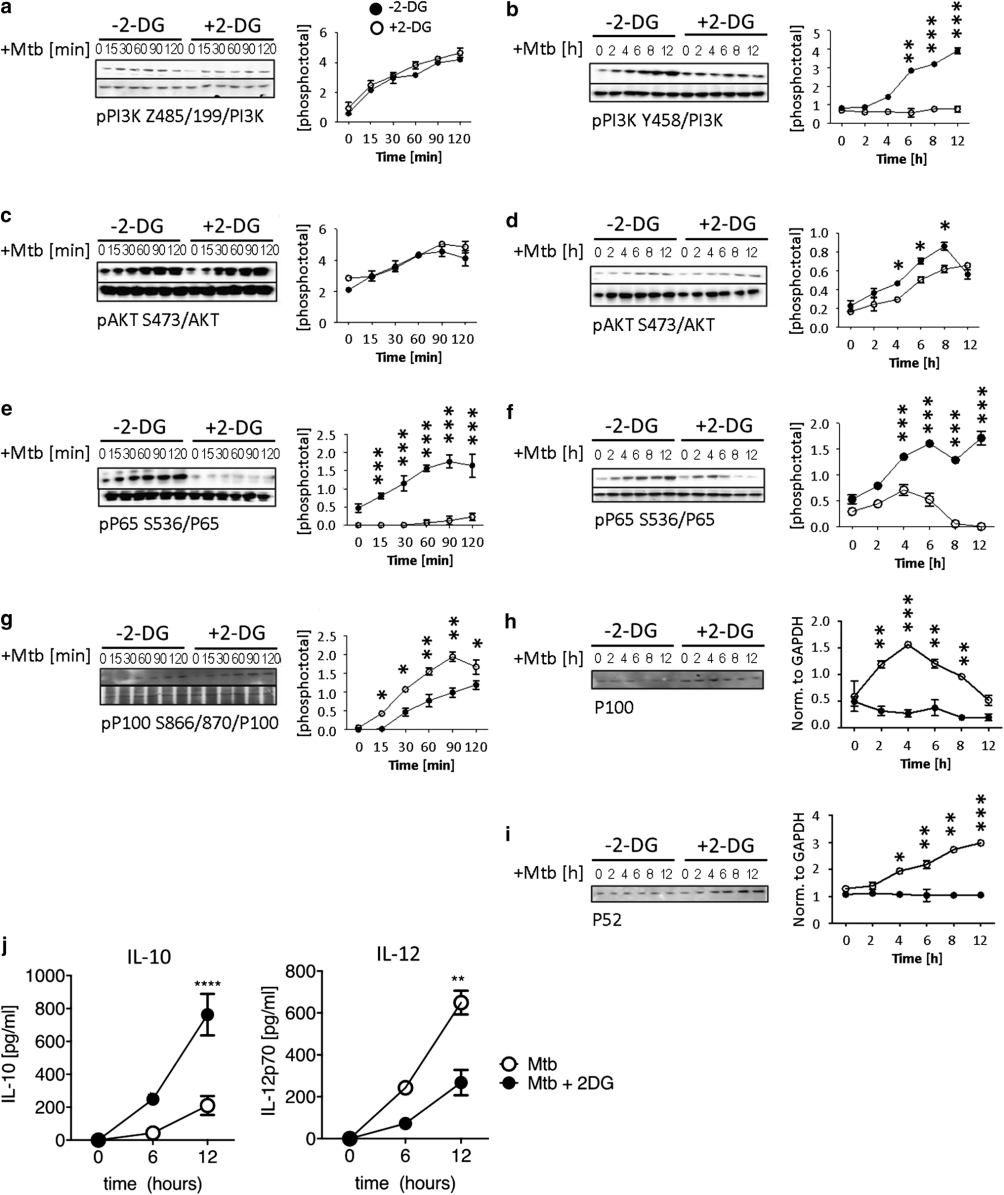
*2-DGtolDC show reduced pro-inflammatory PI3K-AKT and canonical NFκB signalling after Mtb stimulation with increased non-canonical NFκB signalling*. TolDC were prepared as indicated in legend to Fig. 7. Cells were cultured on d6 for 3 h with or without 2.5 mM 2-DG. They were then stimulated with heat-inactivated mycobacterial extract, Mtb (15 μg/ml) for the times shown (0–120 min, or 0–12 h) and assessed for signalling components; immunoblots with corresponding graphical quantifications are shown: **a** Phosphorylation of PI3K-p85 at Y485, 0–120 min with total p85; quantification of PI3K-p85 Y485 0–120 min. **b** PI3K-p85 phosphorylation at 0–12 h; quantification of PI3K-p85 0–12 h. **c** Phosphorylation of AKT at S473 0–120 min, total AKT control is shown; quantification of AKT S473 0–120 min; **d** AKT S473 0–12 h; quantification of AKT S473 0–12 h; **e** NFκB p65 S536 0–120 min and total NFκB; quantification of p65 S536 0–120 min. **f** p65 S536 0–12 h; quantification of p65 S536 0–12 h; **g** Phosphorylation of NFκB p100 S866/870, with total p100 control 0–120 min; quantification of p100 S866/870; **h** Total p100 0–12 h; quantification of p100 relative to GAPDH loading control; **i** Total p52 0–12 h; quantification of p52 relative to GAPDH loading control. 2-DGtolDC are shown in open circles, untreated DC in filled circles. Error bars denote Standard Error of the Mean (SEM), *p* values: * < 0.05, ** < 0.01, *** < 0.001, *n* = 3. **j** 2-DGtolDC show increased IL-10 and decreased IL-12 secretion. Media supernatants were removed at 0, 6 and 12 h post-Mtb stimulation (15 µg/ml), with and without 2.5 mM 2-DG from cells. Supernatant was then assayed via ELISA for IL-10 and IL-12 concentration. *n* = 3/genotype. *p* values: * < 0.05, ** < 0.01, **** < 0.0001

We next asked if sustained pSIRP-1α activity in 2-DG-treated tolDC had a downstream effect on PI3K after stimulation with Mtb. While there were no changes in p85 PI3K phosphorylation within the first two hours of Mtb stimulation (Fig. 8a), we found that Mtb stimulation of non-2-DG-treated tolDC induced high levels of p85 PI3K by 6 h which continued to increase up to 12 h. In contrast, 2-DGtolDC failed to induce any significant level of p85 PI3K (p < 0.001, 2-DG-treated vs. non-2DG-treated tolDC) (Fig. 8b). The further downstream signalling component AKT followed a similar pattern, with no significant change in phosphorylation status within the first 2 h of stimulation with Mtb (Fig. 8c) but a significantly reduced level at 4 h (*p* < 0.05, 2-DG-treated vs. non-2DG-treated tolDC) (Fig. 8d) lasting till 8 h post-stimulation.

Since PI3K and AKT signal downstream to activate NFκB [60], we further investigated the effects of 2-DG on NFkB signalling in tolDC. 2-DGtolDC failed to activate NFkB even when stimulated by Mtb. Stimulation with Mtb induced minimal levels of p-p65 S536 at any time from 2 to 12 h following stimulation with Mtb (*p* < 0.001) (Fig. 8e, f). In contrast, p-p100 was significantly increased in 2-DGtolDC in the first 2 h (*p* < 0.001) (Fig. 8g). We also found that total levels of p100 increased in 2-DG tolDC, but not in untreated tolDC, the levels peaking at 4 h (*p* < 0.001) and followed by a rapid decline (Fig. 8h). There was a concomitant increase in p52 expression from 4 h onwards in 2-DG-treated tolDC, while untreated control tolDC had consistently low expression over the 12 h period (*p* < 0.001) (Fig. 8i). These data show that, while canonical NFκB signalling is repressed in 2-DG-treated DC, non-canonical p100 is activated, and cleaved into transcriptionally active p52.

To demonstrate a downstream effect of 2-DG on cytokine signalling by tolDC we also performed an ELISA analysis of tolDC IL-10 and IL-12 secretion by assaying the supernatants from cultured tolDC. As shown in Fig. 8j, IL-10 secretion by Mtb-stimulated tolDC was markedly increased by exposure to 2-DG while the reverse was true for IL-12.

The data in Figs. 7 and 8, therefore, suggest that sustained, upregulated hyperphosphorylation of SIRP-1α by 2-DG stabilises tolDC function by preventing TLR-induced proinflammatory PI3K and NFκB activation while promoting anti-inflammatory non-canonical NFκB activity and increased IL-10 production.

### A single treatment with 2-DGtolDC protects against experimental autoimmune uveitis (EAU)

Given the stable tolerogenic phenotypic and in vitro immunosuppressive effects of 2-DGtolDC, we next determined if 2-DGtolDC can exert in vivo tolerogenic effects using the inducible EAU murine model of ocular inflammatory disease. IRBP-peptide primed tolDC (30 μg/ml) treated with 2.5 mM 2-DG for 24 h (1 × 10^6^ cells in 100 µl PBS) were injected s.c. into C57BL/6 J mice and compared with untreated IRBP-peptide primed tolDC and PBS vehicle alone. Uveitis was induced 24 h later by immunising mice with IRBP peptide 1–20 (500 µg/mouse in 100 µl), emulsified in CFA containing 3.5 μg/ml Mtb, and simultaneous i.p. injection of PTx. Signs of clinical disease were assessed by fundoscopy [26, 42] at d14, 21 and 28 post-inoculation and histological eye exams performed at d28.

**Fig. 9:**
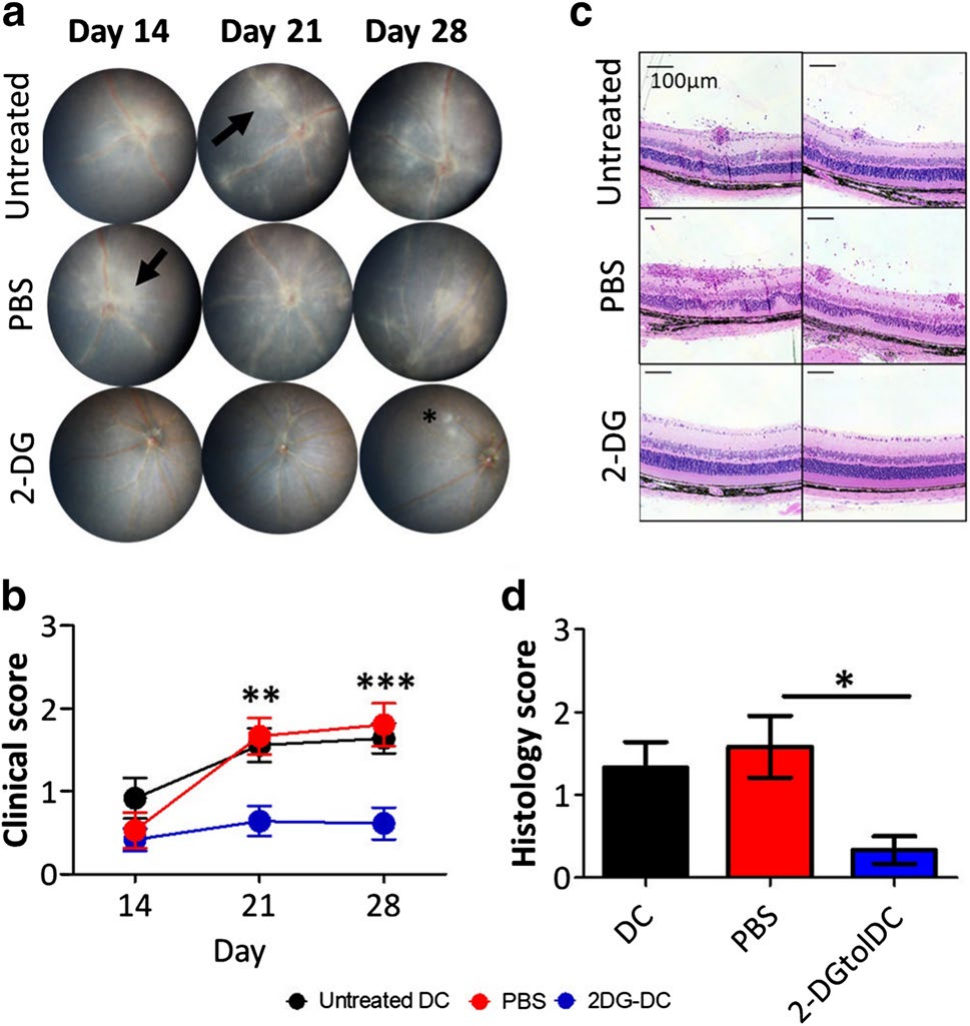
*A single subcutaneous inoculation of mice with 2-DG-treated DC (2-DGtolDC) prevents the development of EAU*. Bone marrow tolerogenic DC (BMtolDC) were prepared as described in Methods and cultured for 24 h with or without 2.5 mM 2-DG in the presence of interphotoreceptor retinol-binding protein (IRBP) peptide (30 μg/ml). 1 × 10^6^ cells suspended in 100 µl of PBS were inoculated subcutaneously (s. c.) to the neck. 24 h later, IRBP peptide (500 µg/mouse) was emulsified in Complete Freund’s Adjuvant (CFA) containing 3.5 μg/ml of Mtb extract, and injected s.c. at the hind limbs. Pertussis toxin (1 μg/mouse) was administered intraperitoneally (i.p.) at the same time. Mice treated with untreated tolDC, 2-DGtolDC and vehicle (PBS) were compared for clinical signs of eye disease. **a** Representative fundus images of mice inoculated with either untreated tolDC, PBS (negative control), or 2-DGtolDC (2.5 mM over 24 h). White sheathing surrounding the vessels indicates widespread vasculitis with onset at d14. Black arrows indicate patches of granulomatous inflammation. Note absence of disease in 2-DGtolDC-treated mice apart from a localised single lesion at d28; **b** Fundus images were randomised and scored by two masked independent graders, according to our previously reported Clinical Score (CS) criteria; see Methods (*n* = 36 mice per group); **c** Representative histology images of the posterior segment of mice eyes 28 days post-inoculation. Mice inoculated with PBS (control) or with untreated DC show evidence of moderate to severe retinal inflammation with vasculitis, retinal folds, granulomas and vitreous cell infiltrates. In contrast, mice treated with 2-DGtolDC have normal retinal morphology. **d** Semi-quantitative masked grading of histological sections (see Methods) (*n* = 6 mice per group), untreated DC (black), PBS (red) and 2-DGtolDC (blue) [26]. Resin embedded sections were stained with haematoxylin and eosin. Scale bar = 100 μm. Error bars denote Standard Error of the Mean (SEM), *p* values: * < 0.05, ** < 0.01, *** < 0.001

Clinical signs of EAU developed in mice injected with either PBS or with untreated tolDC, observed as white retinal infiltrates and vasculitis which increased in severity and extent through d14, 21 and 28. However, mice injected with 2-DGtolDC developed no evidence of disease at d14 and 21, with only occasional small infiltrates evidenced at d28 (Fig. 9a). There was no significant difference in disease severity in mice that received PBS (mean Clinical Score, CS, 1.5) or untreated tolDC (CS, 1.5) at d21 and 28 post-immunisation. However, mice injected with 2-DGtolDC had significantly reduced clinical scores (CS 0.5) (*p* < 0.001: PBS vs. 2-DG-treated) (Fig. 9a, b) at these times.

Histology examination of the eyes at d28 confirmed the fundus findings with evidence of immune cell infiltration, granuloma development and damage to retinal tissue in control (PBS-injected) mice, and in mice given non-2-DG-treated tolDC (Fig. 9c, d). Mean severity grade (see Methods) was 1.5 for PBS-injected mice, and 1.25 for mice given non-2DG-treated tolDC. In contrast, mice given 2-DGtolDC had minimal signs of inflammation with an average histological score of 0.25, no visible granulomas or tissue damage and scarce, scant immune cell infiltrates mostly restricted to the vitreous cavity (Fig. 9c, d). These data showed that a single s.c. injection of 2-DGtolDC was sufficient to prevent induction of EAU, indicating a profound stable (> 28 days, duration of observation) in vivo tolerogenic effect.

A graphical summary of the data in this paper is presented in Supp. Figure 5.

## Discussion

Targeting glucose metabolism in immune cells is an attractive approach to manipulating immunity [61]. In this regard, inhibition of glycolysis has been reported to suppress immune reactions, including autoimmune responses, in experimental animals principally by systemic administration of glycolytic inhibitors such as 2-DG and 3-bromopyruvate [41, 62, 63]. However, since this approach targets several types of immune cells it is difficult to be sure which component(s) of the immune response are modified and so the precise mechanism of action remains obscure. In addition, on a more practical level, daily administration of 2-DG might be difficult to translate to clinical use if only because of potential side effects of long-term administration.

Here, we adopt a different approach using glycolysis inhibition of ex vivo DC to enhance and stabilise their tolerogenic properties prior to adoptive transfer. We describe a procedure based on previous work [17, 33, 45, 64, 65] for preparation of mouse tolDC comprised of a mixed population of myeloid cells containing preDC/tolDC and MDP monocyte/macrophages. Treatment of this cell preparation with low-dose 2-DG (2.5 mM) stabilises an enriched population (up to 60%) of Zbtb46^+^ cells within the major subset of MHC class II^lo/neg^ CD11b^hi^ CD11c^hi^ tolDC (Fig. 2c). Antigen-specific T cell proliferation was reduced in response to activation by peptide primed 2-DGtolDC (Fig. 3) and 2-DGtolDC preferentially secreted IL-10 rather than IL-12 after activation by Mtb (Fig. 8). Remarkably, a single inoculation of 1 × 10^6^ 2-DGtolDC was enough to prevent the development of the inducible murine autoimmune disease EAU for up to 28 days (duration of the experiment), confirming that 2-DGtolDC are stable in vivo and induce a prolonged immunosuppressive effect (Fig. 9).

Glucose metabolism is central to DC function [49]. Basal oxidative phosphorylation (OXPHOS) is the main source of energy for resting DC, undifferentiated DC and tolDC [66] and is regulated by miR-142 [67]. The energy required for conversion of resting, undifferentiated DC to mDC after challenge is underpinned by a metabolic switch from OXPHOS metabolism to anaerobic glycolysis, a process necessary for antigen presentation to T cells [19, 36, 37, 68–71]. In the present study, 2-DG treatment of resting/tolDC downregulated basal OXPHOS metabolism without inducing lactate production (Fig. 5). Challenge of 2-DGtolDC with Mtb also failed to initiate anaerobic glycolysis but instead upregulated an early increase in succinate production thus favouring OXPHOS metabolism (Fig. 6). Furthermore, a second phase of anaerobic glycolysis, seen in untreated tolDC at 120 min after challenge with Mtb, was much reduced in 2-DGtolDC while OXPHOS metabolism occurred in both 2-DG-treated and -untreated Mtb-challenged DC around this time (Fig. 6). These data suggest that tolDC treated with 2.5 mM 2-DG in the presence of 11 mM glucose are programmed preferentially to block anaerobic metabolism and instead sustain OXPHOS metabolism when challenged. Under these conditions, OXPHOS metabolism may simply be the default pathway for handling glucose. Importantly 2-DG, at the optimal concentration of 2.5 mM, did not induce apoptotic cell death in resting or Mtb-challenged tolDC cultured in glucose-containing (11 mM) conditions. Competition for uptake between glucose and 2-DG and for interaction with intracellular hexokinase thus appeared to favour 2-DG since there was potentially four times more glucose available to the cell than 2-DG. Interestingly, 2.5 mM 2-DG not only inhibited Mtb-induced anaerobic glycolysis but prevented glycogen accumulation indicating that glucose metabolism overall was dysregulated in 2-DG-treated cells. It has been shown that intrinsic glycogen stores and glycogenolysis are necessary for full TLR activation of mDC and T cell activation [72] which may thus explain some of the effects of 2-DGtolDC on T cell proliferation reported here. Resting DC treated with 2-DG expressed the surface characteristics of *bona fide* tolDC: *i.e.* low/absent expression of MHC class II, reduced expression of CD86, lack of CD40 expression (Supp. Figure 2a-c and Fig. 1) and close to 100% expression of PDL-1 (Fig. 4). Even when stimulated with Mtb there was no significant upregulation of MHC Class II on 2-DGtolDC unlike that seen with untreated Mtb-challenged tolDC (Fig. 2e). 2-DGtolDC also failed to upregulate CD86 after Mtb stimulation (Supp. Figure 2). These data confirm the stability and “maturation resistance” of 2-DGtolDC.

The effects of 2-DG on glucose metabolism in tolDC as administered here are transient and can be explained by the known half-life of 2-DG. A ratio of glucose to 2-DG in the medium (~ 4:1, see above paragraph) is such that when the effect of 2-DG has declined (0 →  ~ 60 min, short half-life) the cells shift back to full glucose utilisation. In non-Mtb treated, non-activated cells which process glucose through pyruvate into the Krebs cycle and on to OXPHOS, succinate levels are sustained throughout the period in the presence of 11.0 mM glucose (normal conditions for quiescent DC). The competition between 2-DG and glucose for Glut-1 and hexokinase activity means that only partial aerobic glycolysis can be achieved. At the same time, because glucose metabolism is reduced, Krebs cycle metabolism is also reduced for up to 90 min, coinciding with the decline in 2-DG. At 90 min, there is a slight increase in succinate production (1.3-fold, Fig. 5g) but this eventually ceases altogether by 24 h. The storage of glycogen (Fig. 5d) [72] is a known survival behaviour of DC. In Mtb-activated cells, when the glucose flux is increased by increased energy demands [73], 2-DG treatment leads to a marked increase in succinate accumulation (Krebs cycle activity) in the early phase because aerobic glycolysis is reduced to < 70% by 30 min (Fig. 6c). Accumulation of succinate declines as the effect of 2-DG wears off (30–60 min) but increases for both 2-DG-treated and -untreated Mtb-activated DC as the cells revert to normal glucose utilisation (90 min–24 h).

Despite the transient nature of 2-DG on tolDC metabolism in the protocol described here, the effect of 2-DG is to stably re-programme the cells for tolerogenicity. Tolerogenic DC as a potential therapeutic intervention for autoimmune disease and transplant rejection have been studied extensively (reviewed in [74, 75]). Various methods had been explored both experimentally and clinically to generate tolDC including VitD/dex [18–23], inhibition of mTOR [76], retinoic acid treatment [25], activation of the IDO/ARH system [77, 78], LPS/TLR4 ligation/monophosphoryl lipid A [6, 26, 79] and cytokine treatment (IL-10, IL-27, TGFβ (reviewed in [74, 80]). Much work has been done to generate stable DC which would have reduced risk of becoming activated in vivo, currently considered one of the main stumbling blocks to successful tolDC therapy for autoimmune disease [18, 81]. Tolerogenic DC are characterised by expressing reduced levels of one or more of the family of proteins which comprise the transcription factor NFkB, a core inducer of proinflammatory cytokines [82] while direct inhibition of NFkB in DC is currently under investigation as a method of preparing tolDC [7]. However, the precise role of NFkB proteins in DC may be quite subtle and variable in different subsets of DC, since they regulate migratory DC which promote tolerance to self-antigens and immune homeostasis generally [4, 83]. Tissue-resident non-activated sessile preDC/tolDC thus appear to have a basal “constitutive” level of NFkB activity.

Here we show that in vitro 2-DGtolDC express reduced levels of NFkB p65 in response to stimulation with Mtb as well as increased levels of the non-canonical anti-inflammatory p100/p52 transcription factors (Fig. 8h, i). Reduced levels of NFkB may be a downstream effect of inhibition of PI3K/AKT signalling events induced by exposure of DC to 2-DG (Fig. 8a-d) but this would not account for the early onset (15 min) of reduction in NFkB signalling which precedes reduced PI3K/AKT signalling. In fact, the constitutive basal levels of p65 signalling in resting DC is almost completely abrogated simply by the exposure of DC to 2-DG for 3 h (Fig. 8g, h). It would thus appear that reduced NFkB signalling is a direct consequence of sustained 2-DG OXPHOS metabolism and the prevention of anaerobic metabolism (*vide* lactate production) which is characteristic of tolDC as generated through other means (reviewed in [66]).

If NFkB inhibition is a central mechanism for tolDC function, keeping NFkB in check might be important for stabilising and sustaining tolDC activity for instance in the production of IL-10 (Fig. 8j). Tolerogenic DC are characterised by expression of a range of checkpoint molecules including PDL-1 and SIRP-1α. PDL-1 on DC is considered to be a major inducer of T_REG_ via its ligation of PD-1 on T cells and has been shown to inhibit experimental autoimmune encephalomyelitis (EAE) [84]. In the current experiments, PDL-1 expression on pre-DC/tolDC was high both in the percentage of cells and intensity of expression (MFI) and was further increased by treatment with 2-DG (Fig. 4a, b). Moreover, the challenge with Mtb did not decrease PDL-1 expression in 2-DGtolDC.

SIRP-1α is also recognised as a major immune checkpoint molecule on DC [54, 85] although it has also been proposed to have a pro-inflammatory role in a model of colitis [86] and a regulatory role in autoimmune diabetes [87]. SIRP-1α is constitutively expressed on DC presumably by its default binding to various ligands either on adjacent cells (CD47) or to extracellular matrix components such as thrombospondin (for review see [88]). Conventional cDC1 SIRP-1α^+^ cells are required for central tolerance induction [89] while recently it has been shown that human SIRP-1α^+^ cDC2 occupy specific locations in their role in immune surveillance and have been described as the “guardians of the mucosa” [90].

SIRP-1α is phosphorylated in its native state in tolDC (see time point zero, Fig. 7a) and does not require ligation to CD47 to be phosphorylated [91]. It is rapidly dephosphorylated *e.g.* after the challenge of DC with Mtb (Fig. 7a) with extensive loss of surface expression (Fig. 4c). Treatment of DC with 2-DG prevents both these effects (Figs. 4c, 7a). Phosphorylated SIRP-1α is known to sequester AKT [59] which, following activation of PI3K, signals upstream of NFkB [92, 93]. PI3K/AKT signalling is a central signalling pathway in TLR activated DC [92] and culminates in NFkB nuclear translocation and signalling [26]. The sequestration of AKT by pSIRP-1α in 2-DGtolDC would thus stabilise tolDC function by prolonging the inhibition of NFkB and more importantly prevent P65NFkB activation after challenge with, for instance, Mtb. We thus propose that 2.5 mM 2-DG treatment of pre-DC/tolDC enhances and stabilises their tolerogenicity by direct inhibition of NFkB signalling in the immediate phase of antigen/pathogen challenge via inhibition of anaerobic glycolysis while preventing the second phase NFkB activation through sustained pSIRP-1α activity and sequestration of upstream PI3K/AKT.

The effects of 2-DG on the role of PDL-1 in tolDC may be more complex. High expression of PDL-1 is characteristic of tolDC and its expression was increased in 2-DGtolDC (Fig. 4a, b). Immune cells convert phosphoenol-pyruvate to pyruvate during the last step of aerobic glycolysis using pyruvate kinase of which isoforms PKM1 and PKM2 may be active. Immune cells generate large amounts of pyruvate for OXPHOS and for anaerobic glycolysis to supply acute, as well as storage, energy needs mostly using tetrameric PKM1. PKM1 and PKM2 activity, however, are mutually exclusive and PKM2 is considered to be a metabolic sensor responding to glucose starvation [94, 95]. In cancer cells, PKM2 is regulated by protein arginine N-methyl transferase (PRMT), a process which can be prevented by 2-DG. PKM2 exists as a monomer/dimer and translocates to the nucleus to stimulate HIF1α transactivation domain function and recruitment of p300 *HIF1α* and other genes involved in glycolysis, for instance in inflammatory macrophages [96]. PKM2 is also required for expression of LPS-induced PDL-1 in macrophages [97] while activation of PKM2 in DC inhibits activation of Th1 and Th17 cells and suppresses EAE [98] indicating an indirect immunoregulatory role for PKM2. When PKM2 is converted to a tetramer for instance by tetraethyl pyrophosphate (TEPP), it cannot translocate to the nucleus and glycolysis is inhibited [97, 98]. It is, therefore, unclear how PKM2 can orchestrate an immunoregulatory role e.g. via PDL-1 expression on DC while promoting an immunogenic role via ramping up glycolytic activity. This may depend on the timing and context of PKM2 activity and requires further study. With regard to the effects of 2-DG, activation of PKM2 sensitises cancer cells to 2-DG [99] and it is likely that a similar sensitisation occurs in tolDC. Particularly, low-dose 2-DG (2.5 mM) hexokinase II-generated 2-DG6P may be sufficient to permit continuing expression of PDL-1 but insufficient to permit full-throttle *HIF1α* gene expression and lactate production.

Whatever the specific mechanism, the aim of generating protocols for tolDC is to bring this potentially valuable therapeutic intervention to the clinic. Several methods are under investigation in clinical trials including Vit/dex [6, 18], LPS/MPLA [27, 100], as well as direct inhibition of NFkB (liposomes) and inhibition of mTOR as detailed above. Vit/dex treatment of DC induces glycolysis and OXPHOS metabolism and inhibits NFkB while inhibition of mTOR can have pro- and anti-inflammatory effects. None of these methods has shown efficacy in clinical trials, as yet, and the question has been raised as to whether these are feasible treatments [101]. We show here that ex vivo treatment of pre-DC/tolDC using low-dose (2.5 mM 2-DG) generates enhanced stable tolDC which prevents an experimental autoimmune disease (EAU), and this simple approach has potential for ready translation to the clinic.

### Electronic supplementary material

Below is the link to the electronic supplementary material.

Supplementary file1 (PDF 1178 kb)
