# Supplementary material for: Low-dose 2-deoxy glucose stabilises tolerogenic dendritic cells and generates potent in vivo immunosuppressive effects
Source: Cell Mol Life Sci. 2020 Oct 19;78(6):2857–76. doi: 10.1007/s00018-020-03672-y (PMC8004500; doi:10.1007/s00018-020-03672-y)
Supplement: Supplementary file 1 — Supplementary file1 (PDF 1178 kb) [file 18_2020_3672_MOESM1_ESM.pdf]

Low-dose 2-Deoxy Glucose Stabilises Tolerogenic Dendritic Cells and Generates Potent *in vivo* Immunosuppressive Effects

Christofi M[1, #], Le Sommer S[1, #], Mölzer C[1, *], Klaska IP[1], Kuffova L[1, 2], Forrester JV[1, 3, 4, *]

[1]Institute of Medical Sciences, School of Medicine, Medical Sciences and Nutrition, Foresterhill, University of Aberdeen, Aberdeen, AB25 2ZD
[2]Eye Clinic, Aberdeen Royal Infirmary, Aberdeen, Scotland, UK
[3]Ocular Immunology Program, Centre for Ophthalmology and Visual Science, The University of Western Australia, Perth, WA, Australia
[4]Centre for Experimental Immunology, Lions Eye Institute, Nedlands, WA, Australia

[#]These authors contributed equally to this manuscript

[*]*Correspondence*: Professor John V. Forrester (j.forrester@abdn.ac.uk), and Dr. Christine Mölzer (christine.moelzer@abdn.ac.uk), Institute of Medical Sciences, School of Medicine, Medical Sciences and Nutrition, Foresterhill, University of Aberdeen, Aberdeen, AB25 2ZD, Scotland, UK

Supplementary Information

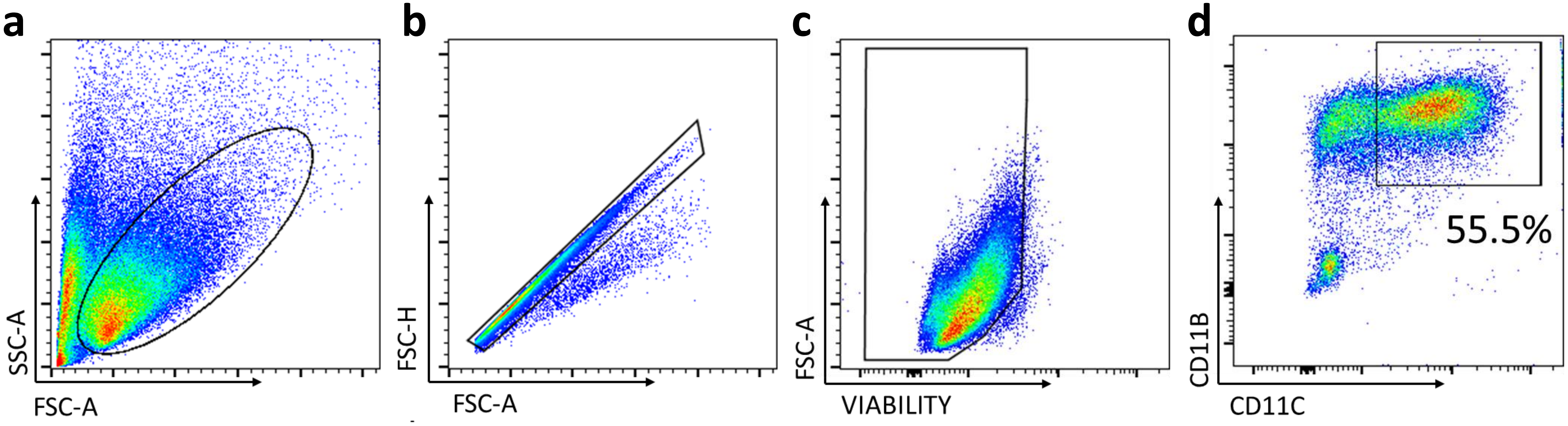

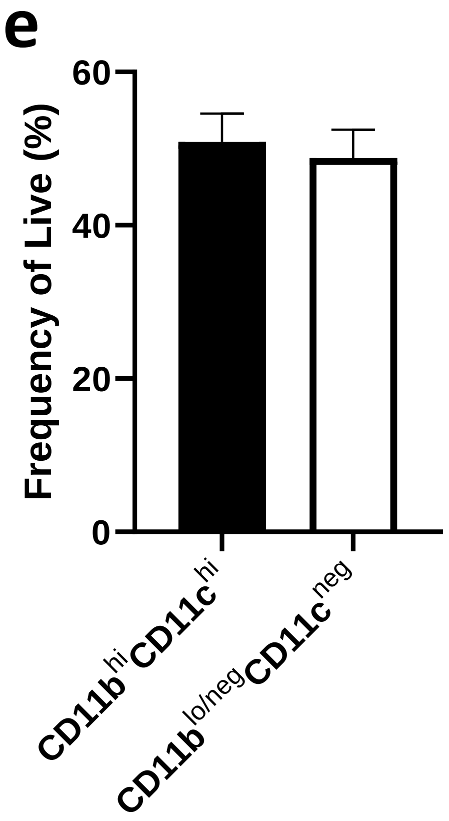

Supp. Fig. 1 *Gating strategy for GM-CSF BMDC cultured cells.* BMDC were depleted of Lin^+ cells (CD4, CD8, CD45R/B220 and MHC II) and cultured in GM-CSF (10 ng/ml) for 6 days with medium exchange every two days. Loosely adherent cell clusters were harvested, depleted of $Gr\text{-}1^+$ cells and resuspended in complete medium (cRPMI). A sample of $1x10^6$ cells was then prepared for flow cytometry (see Methods) and analysed on an LSR II cytometer ($1x10^5$ events recorded). The gating strategy was as follows: *(a)* discrimination of leukocytes by forward scatter vs. side scatter; *(b)* single cells were then selected using a forward scatter height (FSC-H) vs. forward scatter area (FSC-A) pulse geometry gate to discriminate between clumps and single cells; *(c)* live/dead discrimination was achieved using eFluor 455UV fixable viability dye, which stains dead cells, hence the gated negative population corresponds to live cells; *(d)* population of interest ($CD11b^{hi}CD11c^{hi}$) was identified using CD11b vs. CD11c staining within the live-single cell-leukocyte gate; and *(e)* bar chart of the frequency of $CD11b^{hi}CD11c^{hi}$ (black bar) and $CD11b^{lo/neg}CD11c^{neg}$ (white bar) within the live population

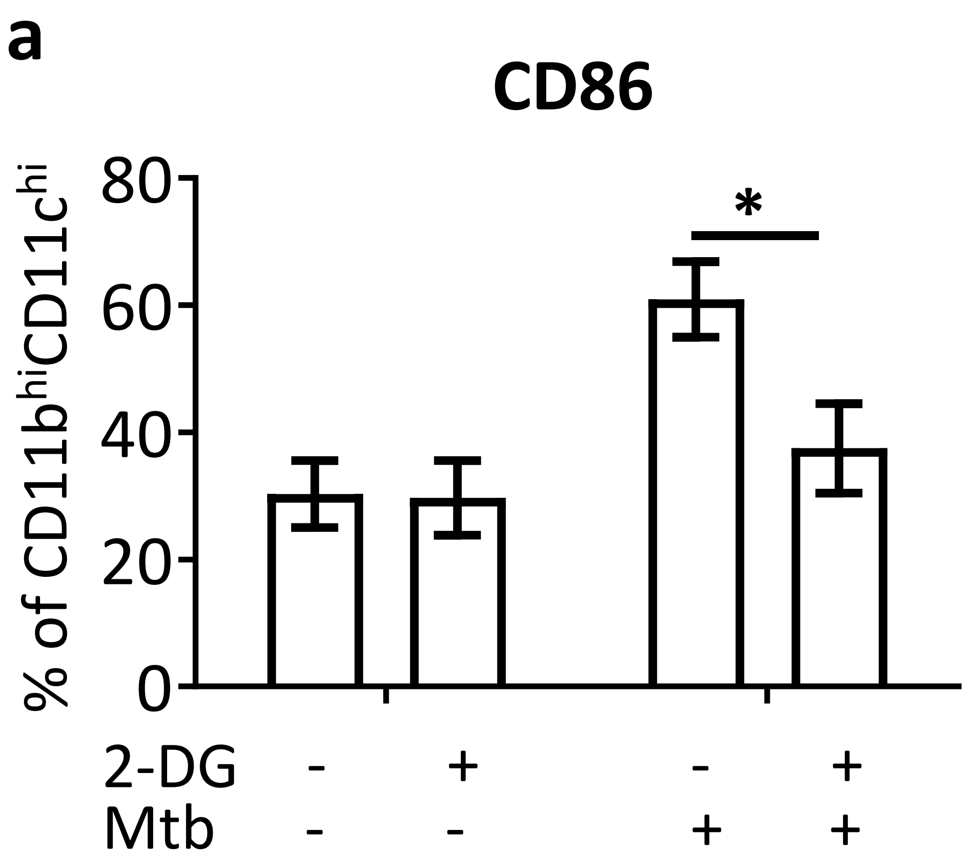

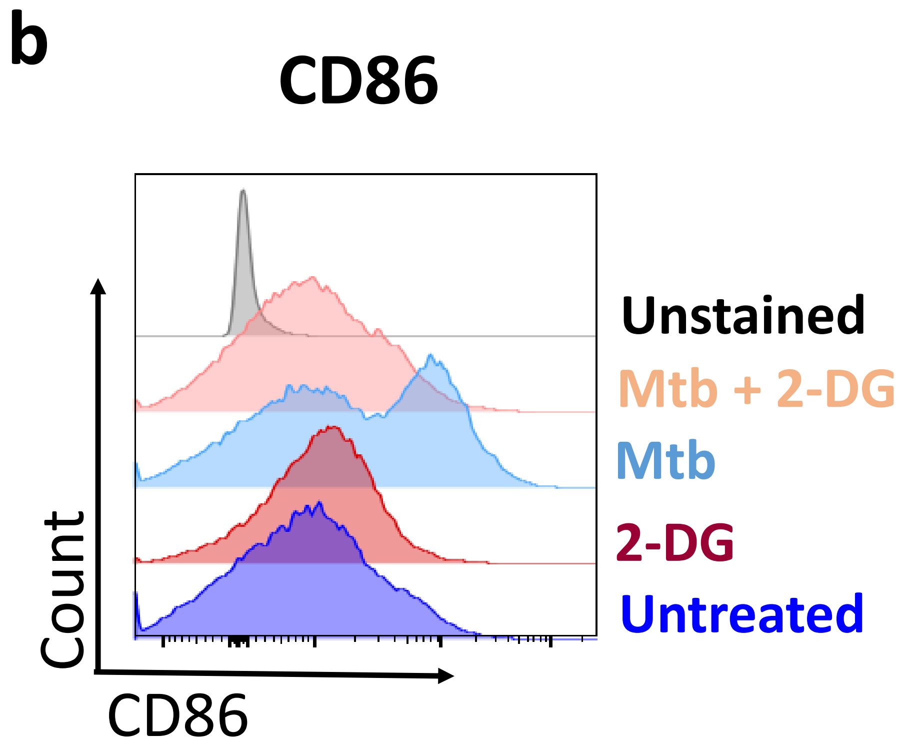

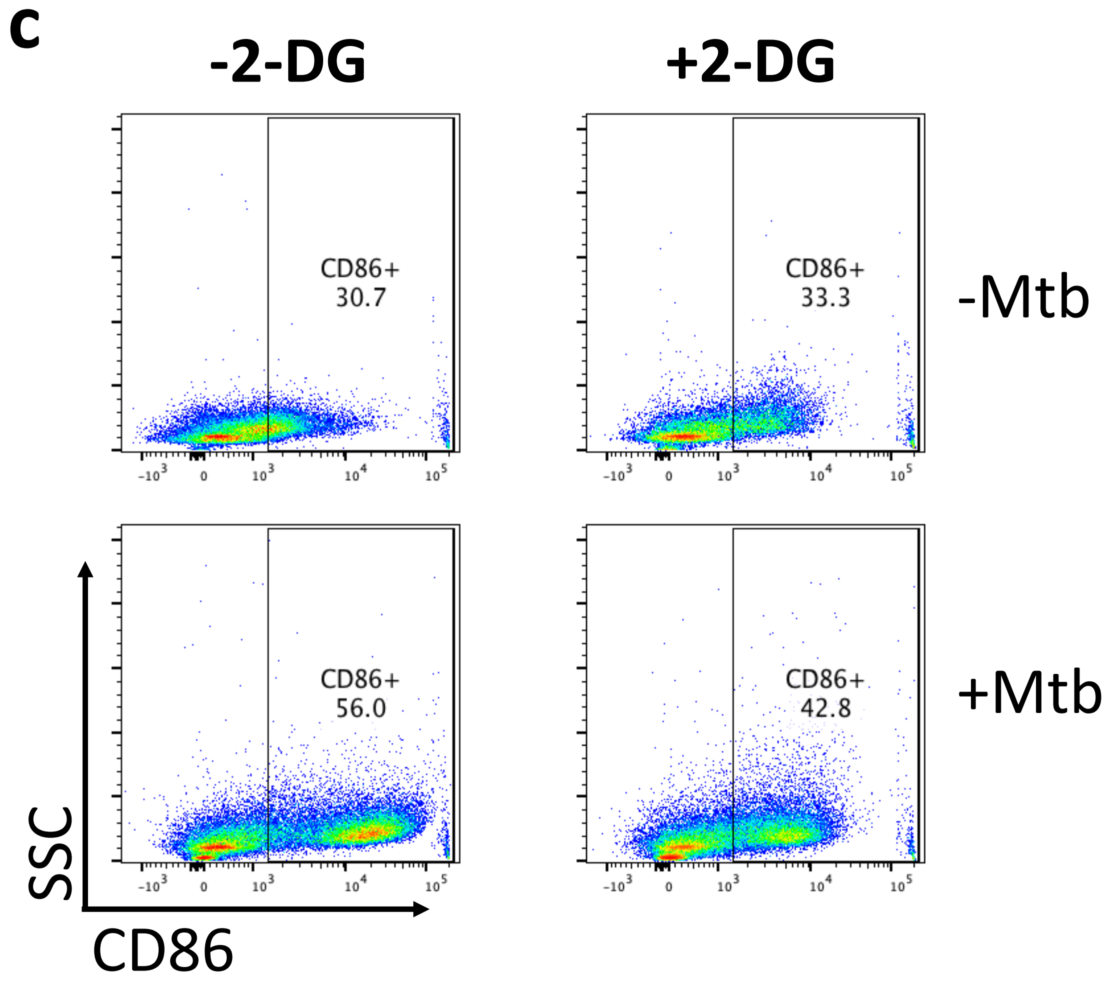

Supp. Fig. 2 *Tolerogenic DC (tolDC) and 2-DG-treated DC (2-DGtolDC) express low levels of CD86; 2-DGtolDC fail to upregulate CD86 when stimulated with Mtb.*
BMDC were prepared as previously described. Flow cytometry was performed to assess CD86 expression on $CD11b^{hi}CD11c^{hi}$ cells (for gating strategy see Supp. Fig. 1): *(a)* percent CD86+ cells within the $CD11b^{hi}CD11c^{hi}$ BMDC population; *(b)* upper panel: representative histograms showing CD86 expression in $CD11b^{hi}CD11c^{hi}$ cells treated with or without 2.5 mM 2-DG, and/or stimulated with Mtb; and *(c)* BMtolDC were cultured as described and treated with 2-DG (2.5 mM) (top panels), and/or stimulated with 15 µg/ml heat inactivated mycobacterial extract, Mtb (bottom panels): representative flow cytometry dot plots of the percentage of CD86+ cells in the $CD11b^{hi}CD11c^{hi}$ population; error bars denote Standard Error of the Mean (SEM), n=3, p value: *<0.05

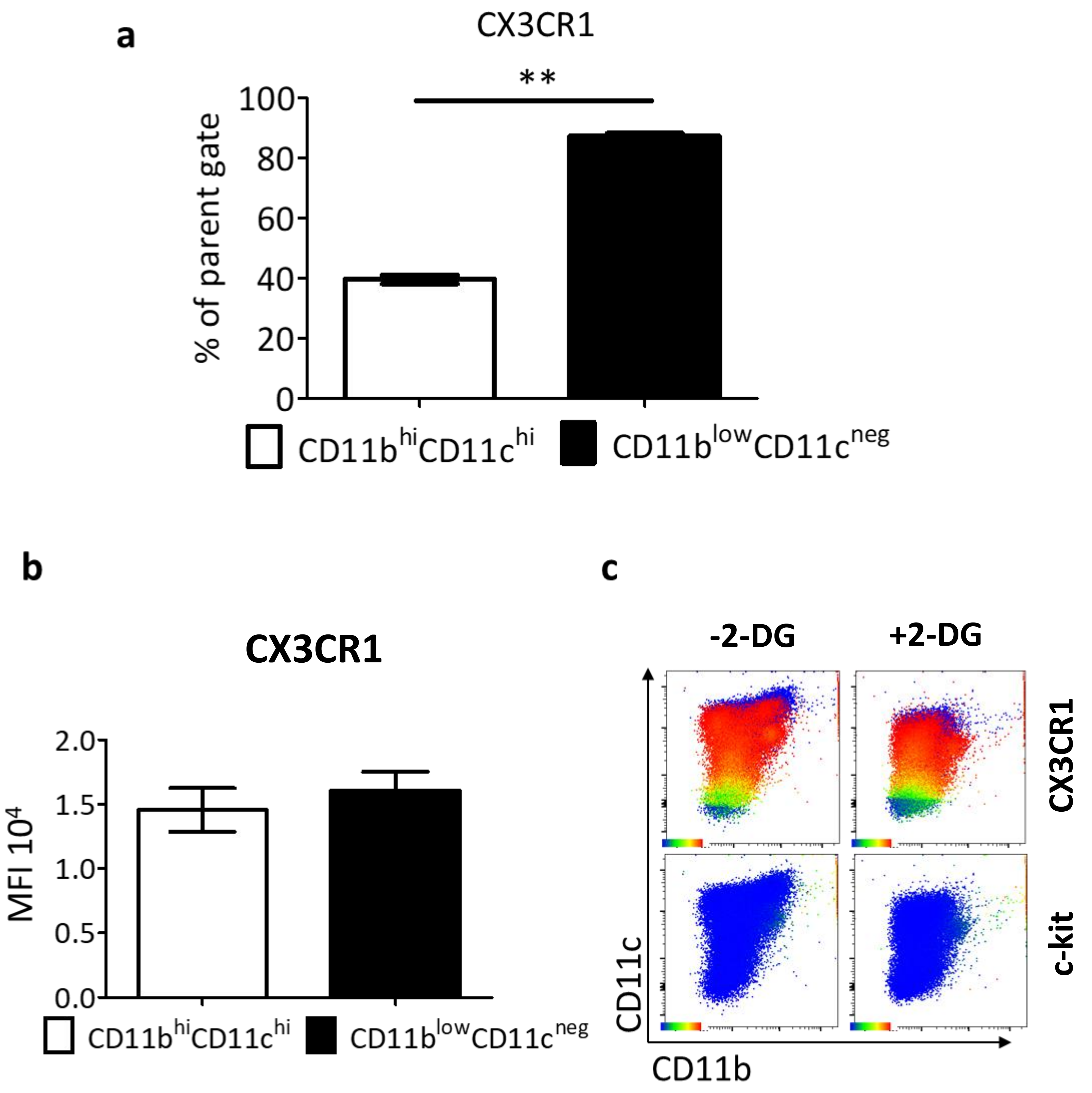

Supp. Fig. 3 *Expression of progenitor markers CX3CR1 and c-kit by GM-CSF-cultured bone marrow cells.* BMDC were prepared as described in Methods and flow cytometry performed to differentiate CD11b^{hi}CD11c^{hi} cells from CD11b$^{lo/neg}$CD11c^{neg} cells (see legend to Supp. Fig 1): *(a)* CD11b^{hi}CD11c^{hi} (presumed DC) expressed moderate levels of CX3CR1 (~40 %) while CD11b$^{lo/neg}$CD11c^{neg} express high levels of CX3CR1 (~80 %). *(b)* CD11b^{hi}CD11c^{hi} and CD11b$^{lo/neg}$CD11c^{neg} CX3CR1 MFI. *(c)* 2-DG treatment slightly reduced the overall percentage of CD11b^{hi}CD11c^{hi} in the BMDC population while increasing the percentage of CX3CR1^{+} cells as shown in representative flow cytometry plots with heat map analysis of CX3CR1 and c-kit expression on non-2-DG-treated DC and 2-DGtolDC. There was minimal c-kit expression with or without 2-DG treatment. Warm colours denote higher relative expression, while cool colours denote lower expression levels

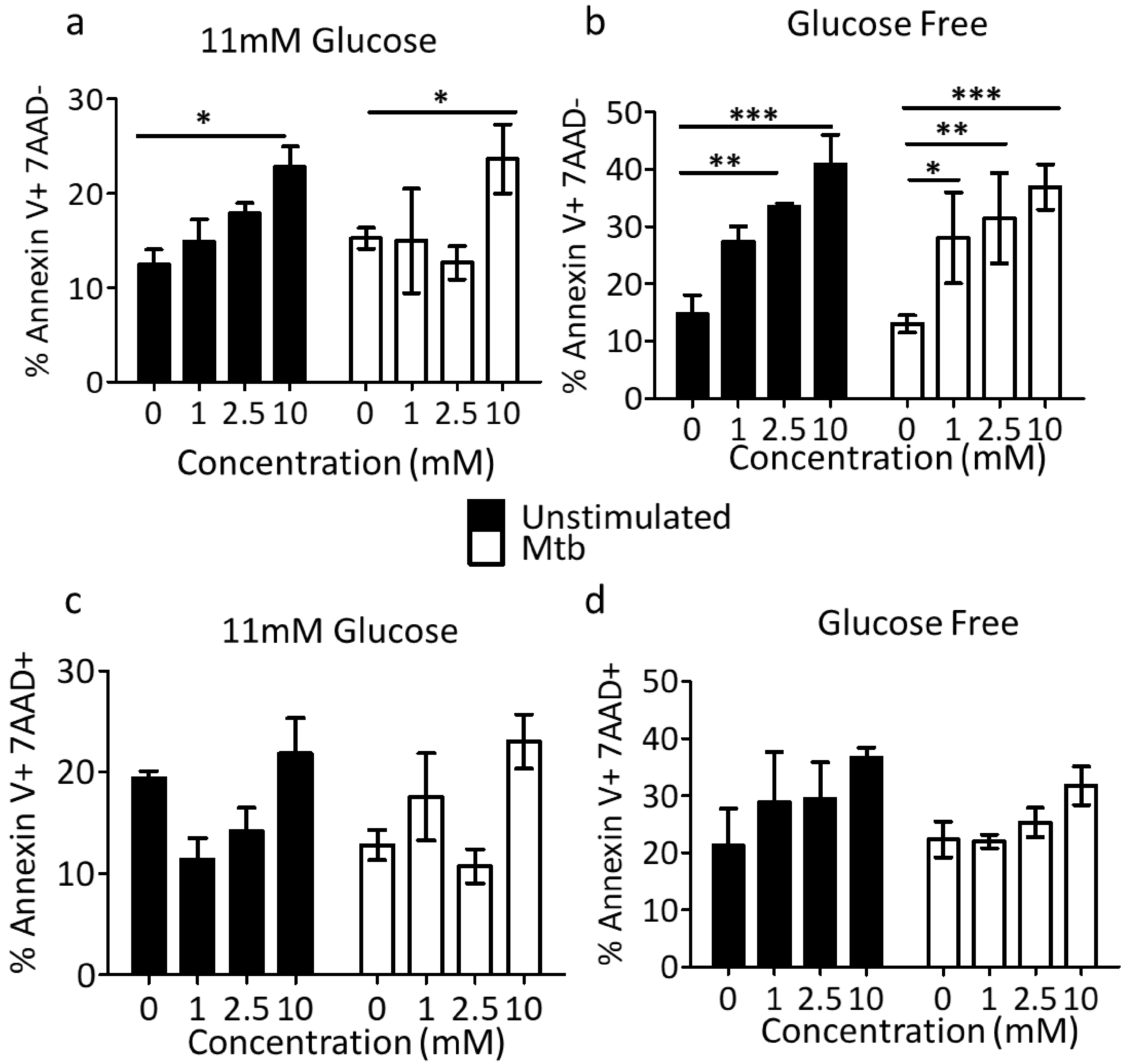

Supp. Fig. 4 *BMDC cultured with 2.5 mM 2-DG in the presence of glucose do not undergo apoptosis.*

BMDC were prepared as described in Methods and on d6 were re-plated in glucose-rich (11 mM) cRPMI media with 2-DG at various concentrations (1, 2.5 or 10 mM 2-DG). Cells were harvested and assessed for apoptosis by flow cytometry using Annexin V staining (see Methods): *(a)* early apoptosis (Annexin V^+ $7AAD^-$ cells) in 2-DG-treated cells, either stimulated with Mtb extract (15 μg/ml) (white bars), or unstimulated (black bars). In glucose rich media there was no evidence of 2-DG-induced cell death up to a concentration of 2.5 mM. At 10 mM, apoptosis levels increased significantly ($p < 0.05$); Mtb stimulation had no additional effect; *(b)* early apoptosis in glucose-free medium; significant levels of cell death were observed particularly when challenged with Mtb antigen**;** *(c)* and *(d)* levels of late apoptosis in BMDC (Annexin V^+ $7AAD^+$ cells) were unchanged in the presence of 2-DG both in glucose-rich (11 mM) and glucose-free media with and without Mtb stimulation. Error bars denote Standard Error of the Mean (SEM); n=3, p values: *<0.05, **<0.01, ***<0.001

Supp. Fig. 5 *Summary of findings* (refer to next page for caption).

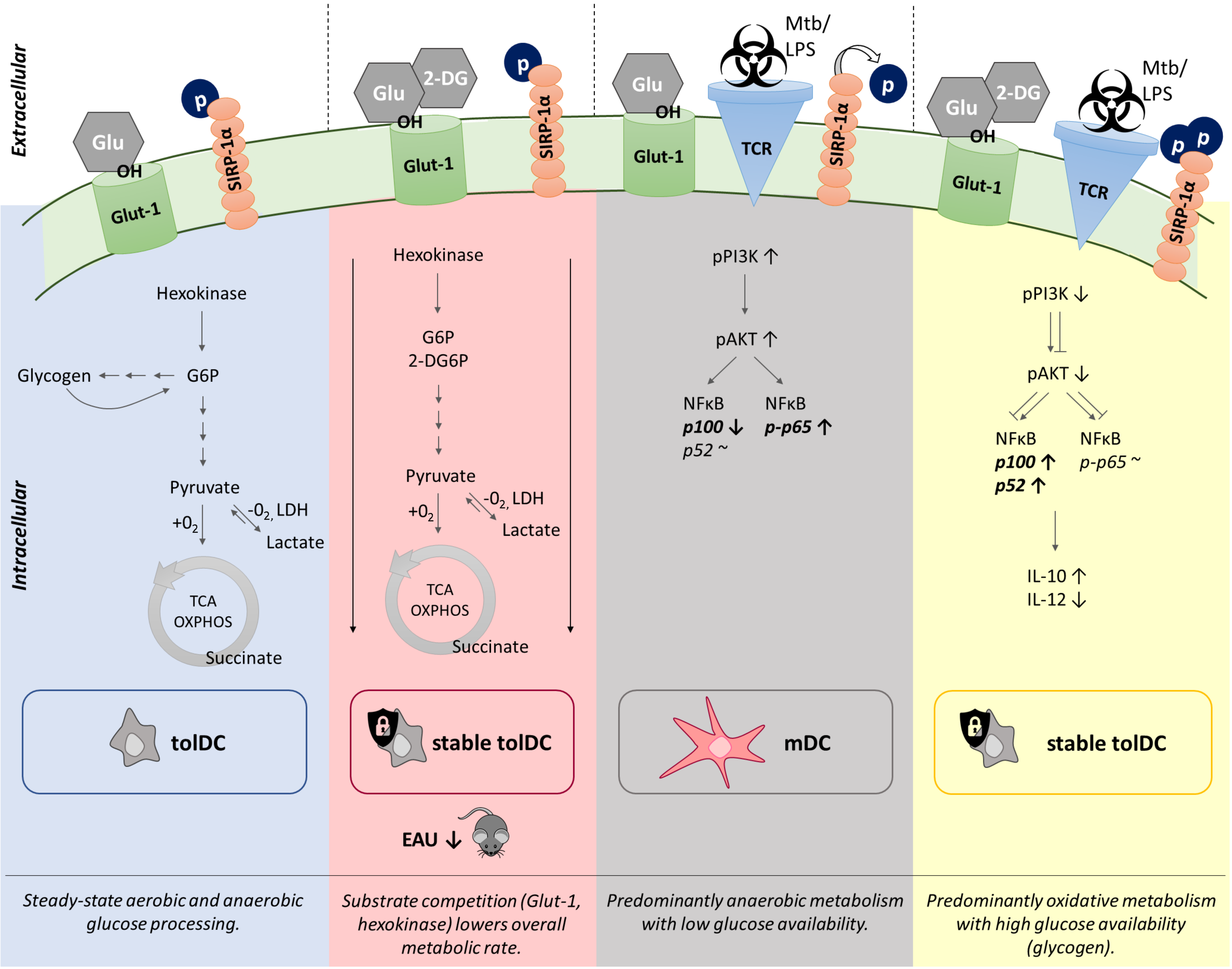

Supp. Fig. 5 *Summary of findings.*

Blue panel: Untreated CD11b⁺ CD11c⁺ Zbtb46⁺ MHCII⁻ tolerogenic DC (tolDC) undergo baseline aerobic and anaerobic glucose metabolism at a steady-state ("resting") rate. In the presence of glucose Glut-1 allows for glucose uptake as is required to meet metabolic demand. SIRP-1α is constitutively activated, i.e. phosphorylated. Untreated tolerogenic DC are unable to halt spontaneous experimental autoimmune uveitis (EAU) in an induced animal model

Red panel: 2-deoxy glucose (2-DG) and glucose compete for uptake through Glut-1 and for intracellular phosphorylation by hexokinase to allow for downstream aerobic/anaerobic substrate processing. This results in an overall reduced metabolic rate. 2-DG stabilises DC in a tolerogenic state, enabling them to prevent EAU progression *in vivo*

Grey panel: Extracellular noxae such as mycobacterium toxin (Mtb) or lipopolysaccharide (LPS) in the presence of glucose induce a metabolic stress response, pushing glucose metabolism towards fermentation (*i.e.* lactate production along with high glucose flux resulting in low substrate availability). This leads to DC activation/maturation (mDC) with increased MHC II surface expression and loss of SIRP-1α phosphorylation. Signalling through NFκB p65 with downstream pro-inflammatory cytokine production aggravates the pro-inflammatory state

Yellow panel: In the presence of both 2-DG and glucose, extracellular Mtb or LPS are unable to activate tolDC. 2-DG exerts a stabilising effect on tolDC, reflected by increased SIRP-1α phosphorylation and signalling through NFκB p100, resulting in an anti-inflammatory state with decreased IL-12 and increased IL-10 expression. These stable tolDC show a high metabolic rate (predominantly aerobic/OXPHOS) with high glucose availability and ample glycogen storage

Abbreviations:
Glu, glucose
2-DG, 2-deoxy glucose
Glut-1, glucose transporter 1
G6P/2-DG6P, glucose-6-phosphate/2-deoxy glucose-6-phosphate
TCR, T cell receptor
LDH, lactate dehydrogenase
LPS, bacterial lipopolysaccharide
Mtb, heat inactivated mycobacterial toxin
OXPHOS, oxidative phosphorylation
TCA, tricarboxylic acid cycle (Krebs cycle)
tolDC/mDC, tolerogenic dendritic cells/mature dendritic cells
EAU, induced experimental autoimmune uveoretinitis
